# The gradient clusteron: A model neuron that learns to solve classification tasks via dendritic nonlinearities, structural plasticity, and gradient descent

**DOI:** 10.1371/journal.pcbi.1009015

**Published:** 2021-05-24

**Authors:** Toviah Moldwin, Menachem Kalmenson, Idan Segev

**Affiliations:** 1 Edmond and Lily Safra Center for Brain Sciences, the Hebrew University of Jerusalem, Jerusalem, Israel; 2 Department of Neurobiology, the Hebrew University of Jerusalem, Jerusalem, Israel; National Research Council, ITALY

## Abstract

Synaptic clustering on neuronal dendrites has been hypothesized to play an important role in implementing pattern recognition. Neighboring synapses on a dendritic branch can interact in a synergistic, cooperative manner via nonlinear voltage-dependent mechanisms, such as NMDA receptors. Inspired by the NMDA receptor, the single-branch clusteron learning algorithm takes advantage of location-dependent multiplicative nonlinearities to solve classification tasks by randomly shuffling the locations of “under-performing” synapses on a model dendrite during learning (“structural plasticity”), eventually resulting in synapses with correlated activity being placed next to each other on the dendrite. We propose an alternative model, the gradient clusteron, or G-clusteron, which uses an analytically-derived gradient descent rule where synapses are "attracted to" or "repelled from" each other in an input- and location-dependent manner. We demonstrate the classification ability of this algorithm by testing it on the MNIST handwritten digit dataset and show that, when using a softmax activation function, the accuracy of the G-clusteron on the all-versus-all MNIST task (~85%) approaches that of logistic regression (~93%). In addition to the location update rule, we also derive a learning rule for the synaptic weights of the G-clusteron (“functional plasticity”) and show that a G-clusteron that utilizes the weight update rule can achieve ~89% accuracy on the MNIST task. We also show that a G-clusteron with both the weight and location update rules can learn to solve the XOR problem from arbitrary initial conditions.

## Author summary

Artificial neural networks (ANNs) have become among the most powerful tools in artificial intelligence and machine learning, enabling computers to solve complex tasks like image recognition. Inspired by the brain, ANNs are composed of simple neuron-like units that perform a weighted sum of their synaptic inputs. Artificial neurons can “learn” by modifying the weight of each input. Biological neurons, however, are more complex. Synapses in a real neuron can interact nonlinearly with each other in a distance-dependent manner due to voltage-dependent mechanisms, such as the NMDA receptor. We created a model neuron, called the Gradient clusteron (G-clusteron) which can use distance-dependent nonlinearities to learn to solve classification tasks by making its synapses attract or repel each other. We tested the G-clusteron on its ability to recognize handwritten digits from the MNIST dataset and showed that it can achieve a high level of classification accuracy (~85%) just by updating its synaptic locations. We also derive a rule for updating the synaptic weights of the G-clusteron and show that having the ability to update both its synaptic weights and synaptic locations allows the G-clusteron to solve the “exclusive or” (XOR) problem, which is famously impossible for a linear neuron.

## Introduction

In the discipline of machine learning, artificial neural networks (ANNs) have gained great popularity due to their impressive success in solving a wide variety of computational tasks [1]. ANNs also hold a special appeal due to their similarity to the networks of neurons in biological brains, lending hope to the possibility that ANNs might serve as a general-purpose framework for replicating human-like cognition. ANNs have also been used by computational neuroscientists to model activity dynamics, computation, and learning processes in the brain [2–6].

Despite their utility for both machine learning and neuroscience, ANNs operate at a level of abstraction that disregards many of the details of biological neural networks. In particular, the artificial neurons in ANNs integrate their inputs linearly; that is to say, each input to a neuron in an ANN is given an independent weight and the neuron applies an activation function to the linear weighted sum of its inputs.

In contrast, synapses in biological neurons display an assortment of nonlinear interactions due to the passive biophysical properties of the cell as well as the active properties of voltage-gated ion channels. One nonlinear channel of particular interest is linked to the N-methyl-D-aspartate (NMDA) receptor. The NMDA channel is both ligand-gated and voltage-gated [7–9], allowing neighboring synapses on a dendrite to interact in a cooperative, supralinear manner. When two nearby NMDA synapses are activated simultaneously, the voltage-dependence of the NMDA receptor creates a voltage perturbation (depolarization) that is greater than the linear sum of the two inputs had they been activated independently [10].

The NMDA receptor has been shown to play a crucial role in structural plasticity, or the growth and elimination of synapses between neurons [11–14]. Structural plasticity plays a crucial role in development and learning [15–19]. Structural plasticity increases the likelihood that temporally correlated synapses are placed next to each other [12, 14, 20–22], which may take advantage of the NMDA supralinearity. While some structural plasticity may be due to new presynaptic-postsynaptic pairs being formed, structural plasticity can allow neurons with extant synaptic connections between them to find the most functionally effective dendritic location to make a synapse. The commonality of multiple synaptic contacts between a single presynaptic-postsynaptic pair of cells, as shown by electron microscopy [23–26], as well as evidence that the number of multiple synapse boutons increases in an enriched environment [27], lends support to the idea that connected neurons may be sampling dendritic locations so as to optimize synaptic placement.

The synergistic coactivation of neighboring inputs on the dendrite via nonlinear NMDA receptors and the ability of a neuron to relocalize its synapses in response to correlated activity have been theorized to have a computational function. An early framework proposed that, by placing synapses with correlated inputs next to each other, a neuron could learn to solve classification tasks. To demonstrate this capability, Bartlett Mel [28] created a simplified model of a neuron with a single dendrite called the *clusteron*. The clusteron’s dendrite consisted of a sequence of discrete locations, from 1 to *N*, where *N* is the number of features in the input (in an image, *N* would be the number of pixels). Each input to the neuron would impinge upon a specific dendritic location. The “activation” of each synapse was defined as the direct input to that synapse multiplied by the sum of the inputs to nearby synapses within a fixed radius on the dendrite.

In order to train the clusteron to recognize a class of patterns (e.g. to identify pictures containing the digit 2), the clusteron is presented with patterns from the class to be recognized (called the positive class) and stores the average activation of each synapse per pattern. At every epoch of learning, the clusteron randomly swaps the locations of “poorly performing” synapses (i.e. synapses with a low activation relative to the other synapses) with each other on the dendrite, eventually resulting in a configuration wherein correlated inputs become spatially adjacent to each other. The adjacent correlated synapses interact nonlinearly with each other, leading to a higher overall activation for the positive class of patterns.

The clusteron model has spurred a wave of research into *synaptic clustering*, the idea that synapses may be selectively bunched together on a dendrite (structural clustering), and that synaptic clusters may receive correlated inputs which can influence a neuron’s computation (functional clustering) [29]. Recent experimental work has provided evidence for both structural and functional clustering [14, 19, 30–33] with implications for visual orientation selectivity [32] and multimodal sensory adaptation [19]. For a review of both experimental and modeling work pertaining to synaptic clustering, see [29].

In this work, we propose an alternative model for learning via dendritic nonlinearities and structural plasticity on the single dendrite, which we call the *gradient clusteron*, or G-clusteron. Unlike the original clusteron model, the G-clusteron uses a dendrite with continuous-valued locations (as opposed to the discrete locations in the clusteron) and a bell-shaped distance-dependent function to model the location-dependence of interactions between synapses. These modifications allow us to analytically derive a gradient descent-based learning rule for the synaptic locations of the inputs to the G-clusteron. We show that the G-clusteron’s location update rule can learn to solve the classic MNIST handwritten digit multiclass classification task with accuracy comparable to logistic regression (i.e. a linear classifier of the form $\hat{y}=sigmoid(w∙x)$, where $\hat{y}$ represents the predicted probability that the input pattern ***x*** belongs to the positive class and ***w*** is a learned weight vector). Moreover, we derive an additional plasticity rule for the synaptic weights of the G-clusteron and show that when the synaptic location update rule and the synaptic weight update rule are used simultaneously, the G-clusteron can learn to solve the exclusive or (XOR) binary classification task, a feat which cannot be accomplished by a linear classifier [34].

## Results

**Fig 1:**
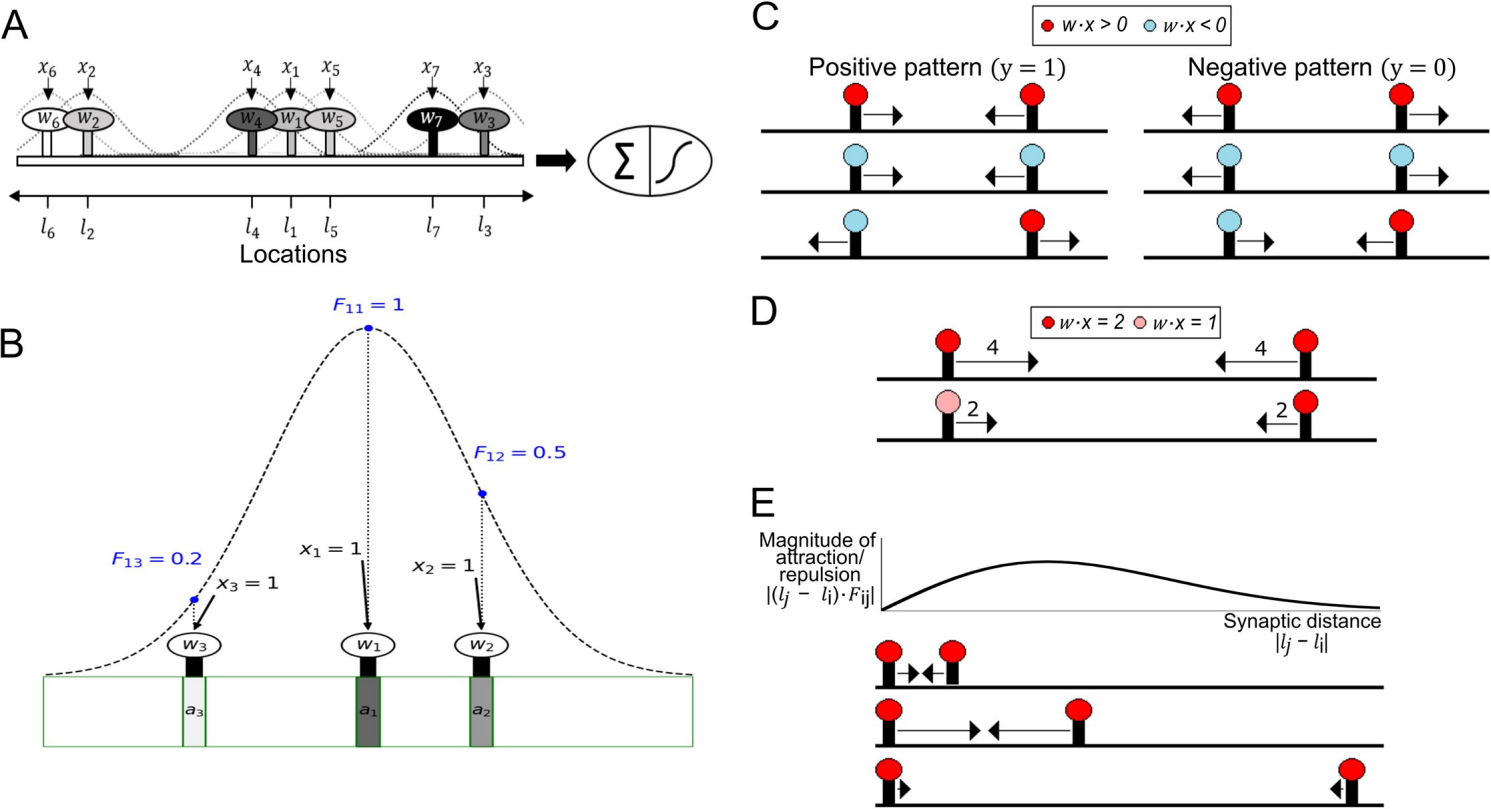
G-clusteron model and synaptic location update rule. (A) Schematic of the G-clusteron. Each input *x*_*i*_ is associated with a synapse at location *l*_*i*_ and weight *w*_*i*_. The activation of each synapse is affected by each other synapse according to a distance-dependent factor (dashed curves). A threshold function is applied to the sum of the activations. (B) Schematic of distance-dependent interactions [Eqs 2 and 3]. The distance-dependent factor between synapse 1 and synapses 2, 3, and itself are shown as blue points along the distance-dependent function (dashed curve). Synapse 1 interacts maximally with itself, moderately with synapse 2, and slightly with synapse 3. Even though the inputs and weights for all synapses are identical, the activations (shaded rectangles, darker shades indicate larger activation) differ due to the relative locations of the synapses (e.g. synapse 1 is centrally located so it is affected by synapses 2 and 3 more than synapses 2 and 3 affect each other). (C) Schematic of the sign-dependence of the location update rule (Eq 7). Colors of the synapses indicate the sign of the weighted inputs (*wx*). For positive patterns (left column), two synapses attract each other if their weighted inputs have the same sign (left column, top and center) and repel each other if the weighted inputs have opposite signs (left column, bottom). In negative patterns this is reversed (right column). (D) Effect of input and weight magnitude on the magnitude of the location update rule. The magnitude of the attraction and repulsion (arrow length) is proportional to |*w*_*i*_*x*_i_*w_*j*_*x*_*j*_|. (E) Top: Magnitude of the location update rule as a function of the distance between two synapses. Bottom: Three examples for the location-dependence of the update rule (aligned to the curve above). Arrow length denotes magnitude of update. This magnitude is multiplied by the weighted input factor of the learning rule from D as well as the magnitude of the error |$\hat{y}-y$| to obtain the final magnitude.

The G-clusteron is a model neuron with a single one-dimensional “dendrite” containing synapses at various dendritic locations (Fig 1A). The *activation* of a synapse is defined as the product of its weighted input with a distance-weighted sum of the weighted input of every synapse on the dendrite (including itself).

Formally, for a given real-valued input pattern vector ***x*** = [*x*_1_,*x*_2_…*x*_*N*_], the real-valued activation, *a*_*i*_, of the synapse with index *i* is:

$$a_{i}=w_{i}x_{i}\sum_{j=1}^{N}f(l_{i},l_{j})w_{j}x_{j}$$ (1)

where *l*_*i*_ is the real-valued location of synapse *i* on the dendrite, *w*_*i*_ is the real-valued weight of synapse *i*, and *f*(*l*_*i*_, *l*_*j*_) is a bell curve-shaped distance-dependent function which determines how much each synapse affects each other synapse, defined as:

$$f(l_{i},l_{j})=e^{\frac{-{\left(l_{i}-l_{j}\right)}^{2}}{r}}$$ (2)

where *r* is a positive-valued “radius” hyperparameter that determines the width of the curve. (From a biophysical standpoint, Eq 2 is analogous to the distance-dependence of the voltage response of a passive cable to an instantaneous current impulse [35]. In this framework, *r* can be thought of as the square of the cable length constant λ, where $\lambda^{2}=\frac{r_{m}}{r_{a}}$, i.e. the ratio of the membrane resistance *r*_*m*_ (in Ω∙*cm*) to the axial resistance *r*_*a*_ (in Ω/*cm*).)

Note that *f*(*l*_*i*_, *l*_*j*_) = 1 when *l*_*i*_ = *l*_*j*_ (i.e. when synapses *i* and *j* occupy the same location) and that *f*(*l*_*i*_, *l*_*j*_) approaches 0 as synapses *i* and *j* move further away from each other (Fig 1B). Also note that *f*(*l*_*i*_, *l*_*j*_) = *f*(*l*_*j*_, *l*_*i*_). For convenience of notation and computation, we define a matrix *F* such that

$$F_{ij}=f(l_{i},l_{j})$$ (3)

*F* is thus a symmetric matrix with ones on its diagonal.

For a given pattern of presynaptic inputs ***x***, the G-clusteron sums its activations together with a bias term *b* to produce:

$$h(x)=\sum_{i=1}^{N}a_{i}-b$$ (4)

Because we will be using the G-clusteron as a binary classifier, we apply a sigmoidal nonlinearity *g* to *h*(***x***) and produce an output $\hat{y}$ ranging between 0 and 1:

$$\hat{y}=g(h(x))$$ (5)

As in logistic regression, $\hat{y}$ can be interpreted as a probability estimate for the binary label for the input pattern ***x***, with values closer to 0 representing a prediction for the label 0 (called the negative class) and values closer to 1 representing a prediction for the label 1 (called the positive class).

The G-clusteron thus differs from the original clusteron [28] in two important ways: 1) each synapse has a real-valued location on the dendrite instead of an integer-indexed location and 2) each synapse’s activation function depends on the inputs of its neighbors as a gradually decreasing distance-dependent function as opposed to a hard cutoff at a fixed distance.

Defining the output of the G-clusteron in this fashion allows us to derive a gradient descent plasticity rule for the synaptic locations (assuming a logit or cross-entropy error loss function, see **Methods**). For each input pattern presented to the G-clusteron, we update each synaptic location according to the rule:

$$l_{i}\leftarrow l_{i}+\Delta l_{i}$$ (6)

where Δ*l*_*i*_ is the update to the location of synapse *i* defined as:

$$\Delta l_{i}=-\eta_{L}(\hat{y}-y)\sum_{j=1}^{N}(l_{j}-l_{i})F_{ij}w_{i}x_{i}w_{j}x_{j}$$ (7)

where *y*∈0,1 is the true label (negative or positive class) for pattern ***x***, and *η*_*L*_ is the positive-valued learning rate for the synaptic locations. This gradient rule for each synapse can be understood as summing over “forces” that depend on pairwise interactions between that synapse and each of the other synapses on the dendrite. The interaction between two synapses depends both on the weighted inputs of the synapses (*w*_*i*_*x*_*i*_ and *w*_*j*_*x*_*j*_) and the distance between the synapses in the following manner (see **S1 Text** for further explanation):

1. For positive-class training patterns (*y* = 1), synapses with same-sign weighted inputs (i.e. *sign*(*w*_*i*_*x*_*i*_) = *sign*(*w*_*j*_*x*_*j*_)) exhibit “attraction” while synapses with opposite-sign weighted inputs (i.e. *sign*(*w*_*i*_*x*_*i*_) = −*sign*(*w*_*j*_*x*_*j*_)) exhibit “repulsion” (Fig 1C, left).
2. For negative-class training patterns (*y* = 0), this trend is reversed: same-sign synapses are repelled, while opposite-sign synapses attract (Fig 1C, right).
3. The magnitude of attraction and repulsion between two synapses is proportional to the product of the weighted inputs of the two synapses (Fig 1D).
4. The magnitude of attraction and repulsion between two synapses is distance-dependent. The attractive/repulsive force is small at very small distances, becomes larger at intermediate distances, and shrinks again at large distances (Fig 1E).

The magnitude of the update for each synapse is also scaled by the magnitude of the error term, |$\hat{y}-y$|.

Eq 7 can be interpreted as a vector field along the dendrite, like force fields in physical systems of particles. If we consider a “unit synapse” such that *wx* = 1 at an arbitrary location *l* on the dendrite, the magnitude and direction of the “force vector” created by the plasticity rule at that location for a given input pattern ***x*** is given by:

$$\Delta l=-\eta_{L}(\hat{y}-y)\sum_{j=1}^{N}(l_{j}-l)f(l,l_{j})w_{j}x_{j}$$ (8)

This interpretation of the location plasticity rule can provide some intuition as to how such an algorithm might be implemented biologically. We can imagine extracellular chemical signals being released at regions of the dendrite where there is a concentration of synaptic activity. Excitatory and inhibitory inputs would have different chemical signals associated with them. The diffusion of these chemicals in the extracellular space around the dendrite would create a location-dependent field of chemical gradients. These chemical gradients could induce presynaptic axons to form or eliminate synapses on the dendritic regions that were recently active, with differential effects for excitatory and inhibitory synapses.

### Toy examples

To illustrate how the location update learning rule works, we consider several toy problems. For these problems, instead of training the G-clusteron to discriminate between two classes, we will show what happens when the G-clusteron is given a dataset where all examples are from the positive class (in other words, the G-clusteron is tasked with maximizing its output on all the input patterns, see **Methods** for an explanation of how the error signal $\hat{y}-y$ is implemented in these tasks) and when all examples are from the negative class (so the G-clusteron must minimize its output for all input patterns). For all the examples in this section, all synaptic weights are fixed to have the value 1 and don’t change over the course of learning; our results thus strictly depend on the synaptic locations and input values.

**Fig 2:**
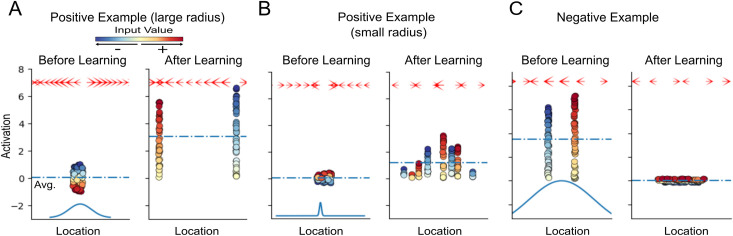
Dynamics of synaptic clustering due to the G-clusteron update rule. (A) Training the G-clusteron on a single positive pattern (see S1 Movie). Synaptic input values (colored circles) were drawn uniformly between -1 and +1. Position of circles along the X-axis denotes synaptic location on dendrite; their location along the Y-axis denotes synaptic activation value. In the initial epoch (left), synaptic locations are randomly initialized in close proximity. During learning, synapses with positive inputs (*x*>0) move in the opposite direction from synapses with negative inputs (*x*<0). In the final epoch (right) separate clusters are observed for positive and negative inputs. Dashed blue line shows the average synaptic activation, which in this case is increased by the plasticity rule. Arrows at the top of each panel denote the magnitude and direction of the “force field” created by the plasticity rule (Eq 8); by convention, the arrow at each location points in the direction that a unitary positive input (*wx* = 1) would move according to the plasticity rule. Blue curve at the bottom of the left panel shows the width of the distance-dependent function (dependent on the radius hyperparameter *r*) for this example. (B) Same as A with a smaller value for *r* (see S2 Movie). Here the plasticity rule operates on a more local scale, creating multiple smaller same-sign clusters instead of two larger clusters as in A. (C) Same as A for a negative pattern (see S3 Movie). Before learning, synapses are initialized in separate clusters for positive and negative inputs (left). Over the course of the learning, synapses are pulled toward each other until they are intermixed (right). Note that after learning the average activation level decreases.

For the first toy problem, we create a single synaptic input vector ***x*** where the values of ***x*** are randomly distributed between -1 and 1. This input vector is repeatedly presented to the G-clusteron with a positive label (*y* = 1), such that the G-clusteron will attempt to maximize its overall activation. Because the interaction between synapses in Eq 1 is multiplicative, a sensible strategy would be to minimize the distance between synapses with same-sign inputs and maximize the distance between synapses with opposite-sign inputs. This would result in two clusters on the dendrite: one with positive-valued synaptic inputs and one with negative-valued synaptic inputs. The location update rule, by causing attraction between same-sign synapses and repulsion between opposite-sign synapses, does exactly this (Fig 2A and **S1 Movie**).

We now show how the radius hyperparameter *r* (i.e. the width of the distance-dependent curve) affects the learning rule for input patterns from the positive class. In the first example, *r* was sufficiently large relative to the initial dispersion of synapses such that all the synapses could “feel” each other, thus enabling all synapses with the same sign to eventually aggregate into a single cluster. However, if *r* is reduced, synapses that start further apart from each other do not exert a strong pull on each other, so instead the learning rule operates in a more local fashion, creating several same-sign clusters (Fig 2B and **S2 Movie**).

We next train the G-clusteron on the same input pattern, but this time with a negative label (*y* = 0), such that the G-clusteron will minimize its overall activation. Here we pre-initialize the G-clusteron in a high-activation state where synapses start in separated homogenous-signed clusters. Inversely to the first strategy, we would like opposite-sign inputs to become intermixed. Because the plasticity rule flips attraction and repulsion for input patterns from the negative class, we achieve the expected result (Fig 2C and **S3 Movie**).

Having demonstrated that the G-clusteron learns by aggregating and dispersing same-sign and opposite-sign synaptic inputs for individual input patterns, we now illustrate that this mechanism serves to learn the correlation structure of a dataset comprised of multiple input patterns. To this end, we create a 20-dimensional multivariate Gaussian distribution where each dimension of the Gaussian had a mean of 0 and a covariance matrix Σ such that:

$$\Sigma_{ij}=\{\begin{array}{c} 1\\ 0.7\\ -0.2 \end{array}\begin{array}{c} if\;i=j\\ if\;i\neq j\;and\lceil i/5\rceil =\lceil j/5\rceil \\ otherwise \end{array}$$ (9)

**Fig 3:**
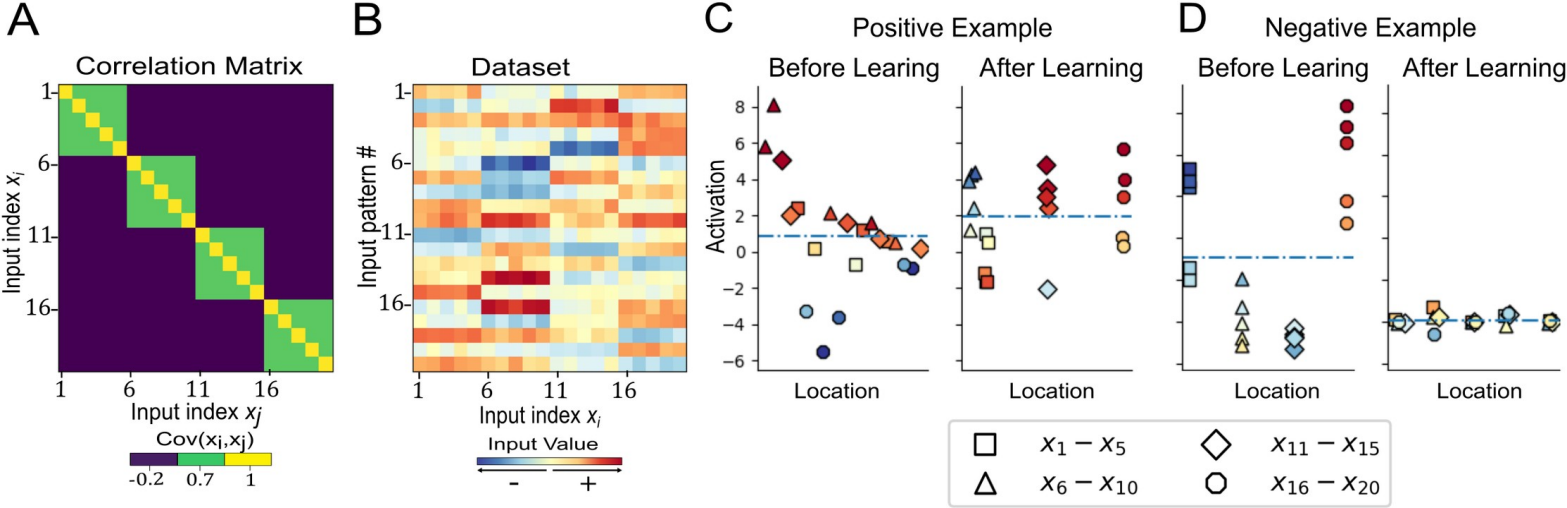
Dynamics of synaptic clustering for a multivariate Gaussian dataset with correlated input dimensions. (A) Covariance matrix for multivariate Gaussian example (Eq 9). This covariance matrix generates samples where dimensions *x*_1_…*x*_5_, *x*_6_…*x*_10_, and so on are positively correlated with each other, while all other correlations are negative. (B) Patterns drawn from the multivariate Gaussian defined with the covariance matrix in A. Each row is an input pattern presented to the G-clusteron. (C) The G-clusteron is initialized with randomized synaptic locations (left) and presented with positive-labeled input patterns as in B (see **S4 Movie**). Correlated inputs have the same shape; colors indicate input values for the pattern (as in B) presented in the depicted epoch of the algorithm. To increase activation, the G-clusteron groups together correlated inputs (right). (D) Same as C but for a negative example (see **S5 Movie**). For illustration, the G-clusteron is initialized with correlated inputs clustered together (left), as in the right panel of C. Over the course of learning the synapses eventually form clusters of negatively correlated synapses (right).

In other words, dimensions *x*_1_…*x*_5_ were all positively correlated with each other, as were dimensions *x*_6_…*x*_10_, *x*_11_…*x*_15_, and *x*_16_…*x*_20_, but otherwise, dimensions were slightly negatively correlated (Fig 3A). As such, each vector sampled from this distribution would exhibit similarity between the first five dimensions, second five dimensions, and so forth (Fig 3B).

As the mean of each dimension is 0, a linear neuron (without a bias) would be unable to increase its average output on this dataset, as there is no independent information in each synapse that could be given a linear weight. However, the G-clusteron can take advantage of the correlational structure of the data. Because the groups of positively correlated inputs will tend to all be either all-positive or all-negative in any given input pattern, the learning rule will gradually move the positively correlated synapses together, creating dendritic clusters of synapses with correlated inputs. As these clusters form, the output of the G-clusteron increases (Fig 3C and **S4 Movie**). Inversely, the G-clusteron can learn to decrease its output on this dataset by clustering negatively correlated inputs (Fig 3D and **S5 Movie**). We verified that the G-clusteron appropriately increases or decreases its average activation for this task by running 100 trials of the algorithm and checking the average activation before and after learning (See **S2 Text**). The average activation on the positive class task before learning was 0.86, and after learning was 2.95 (average signed d’ = 1.095). For the negative class task, the average activation before learning was 2.03, and after learning was 0.44 (average signed d’ = −1.155).

### Learning MNIST

**Fig 4:**
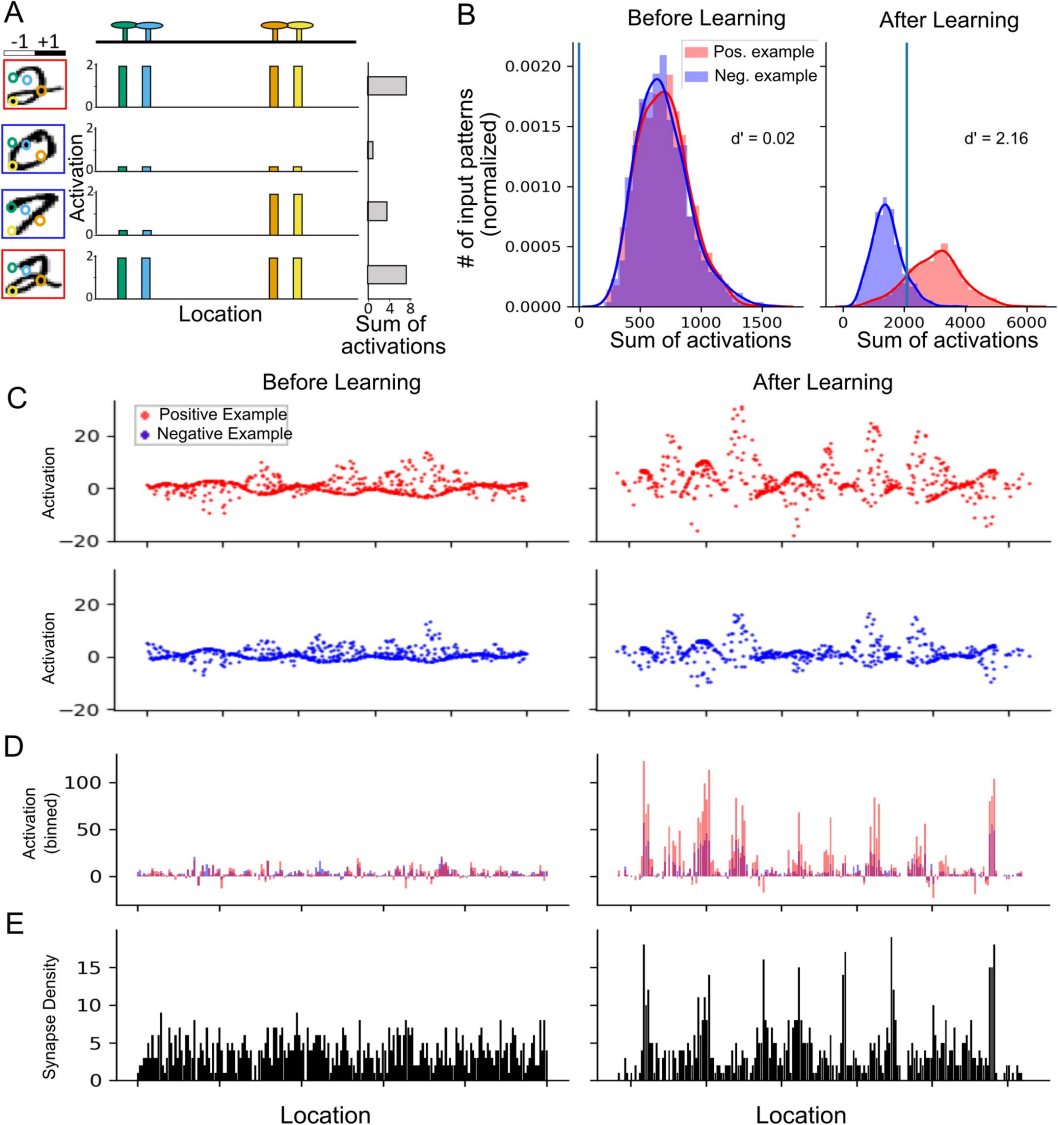
G-clusteron classification of handwritten digits (MNIST) and the resultant distribution of synaptic locations and activations. (A) Schematic of G-clusteron performing the one-versus-all classification task, where it must produce a larger activation for images containing the positive class 2 (digit with red border) than any other number (digit with blue border). Colored circles in the digit images indicate exemplar pixel locations mapped to exemplar synapses on the dendrite with the same color, generating two dendritic clusters (green-blue and orange-yellow, respectively). The synapses within these clusters will be maximally activated if both inputs to the cluster are the same sign (i.e. both black or both white), while opposite sign inputs will result in lower activation. (B) Exemplar histogram of sum of synaptic activations from the G-clusteron tasked with classifying the digit 2 (label *y* = 1, red) versus all other digits (label *y* = 0, blue) before learning (left) and after learning (right). Over the course of learning, the G-clusteron increases its activation on images containing a 2 relative to images displaying other digits, enabling binary classification. Blue vertical line is the value of the bias term. (C) Synaptic activations for positive (top) and negative (bottom) patterns before (left) and after (right) learning. Note the increase in synaptic activations for positive patterns during learning. (D) Sum of the activations within small bins of dendritic length averaged over positive (red) and negative (blue) datasets before and after learning (aligned with C). Note that there are small regions (functional clusters) where the activations for positive patterns is much larger than for negative patterns. (E) Synaptic density per bin (same for positive and negative input patterns, aligned with C-D). Note that there are several high-density regions (structural clusters) that sometimes correspond to the functional clusters.

The ability of the G-clusteron to learn the correlational structure of a dataset enables it to perform supervised classification by aggregating correlated inputs from the patterns where *y* = 1 and disaggregating correlated inputs from the patterns where *y* = 0 (Fig 4A). We trained the G-clusteron on the MNIST dataset of images of handwritten digits [36] and compared its performance to that of the original clusteron from [28] as well as to logistic regression. Before attempting the all-versus-all classification task, we test the G-clusteron separately on each digit in a one-versus-all classification paradigm (example shown in Fig 4B). For each digit from 0–9, a G-clusteron was trained on a dataset where half of the images were of that digit (positive class, label *y* = 1) and half of the images contained other digits (negative class, label *y* = 0). The G-clusteron was then tested on a holdout test set (See **Methods**). This procedure was repeated for the original clusteron (only positive-class training examples are used for the original clusteron, see **Methods**) as well as logistic regression. The learning and testing process was repeated 10 times for each classifier to ensure performance stability. The results are shown in Table 1.

**Table 1:** Accuracies of one-versus-all on the MNIST handwritten digit dataset. Values are averaged over ten runs, with standard deviations in parentheses.

	0	1	2	3	4	5	6	7	8	9
Clusteron	92.8 (0.8)	83.6(5.3)	82.5(0.5)	85.0(0.5)	77.6(1.7)	74.4(1.5)	87.5(0.8)	87.8(0.8)	80.2(1.1)	81.3(1.0)
Gradient Clusteron	96.8(0.4)	93.9(4.4)	87.6(5.3)	88.5(0.9)	91.8(1.1)	80.7(4.1)	95.1(0.4)	90.1(5.0)	82.3(4.3)	85.8(3.6)
Logistic Regression	98.2(0.0)	98.3(0.0)	94.4(0.0)	93.3(0.0)	96.7(0.0)	91.6(0.0)	96.5(0.0)	95.4(0.0)	91.2(0.0)	92.3(0.0)

For all digits, all three classifiers achieved a classification accuracy far above chance level of 50%. Depending on the particular digit, the clusteron achieved between 74.4% and 92.8% accuracy, the G-clusteron achieved between 80.7% and 96.8% accuracy, and logistic regression achieved between 91.2% and 98.3% accuracy. We emphasize that both the clusteron and the G-clusteron had all their weights fixed to 1 during the entire learning procedure and were only able to update their synaptic locations. Our results on the one-versus-all MNIST task thus demonstrate the remarkable efficacy of structural plasticity-based learning and suggest that the G-clusteron algorithm may be superior to the original clusteron algorithm for solving certain tasks.

The distribution of synapses and synaptic activations before and after learning MNIST (Fig 4C–4E) can be instructive in understanding how the G-clusteron operates. In many instances, over the course of learning, the synapses on the dendrite moved from an approximately uniform synaptic density to a more clustered structure, with some regions of the dendrite having a higher synaptic density than others (Fig 4E). These higher density areas occasionally had larger activations for patterns from the positive class than for patterns from the negative class (Fig 4D), suggesting that the G-clusteron may be building structural clusters to take advantage of correlated inputs in the positive class relative to the negative class. However, not all high-density clusters showed high activation, and some high-activation regions were low density. Thus, while the G-clusteron learning algorithm does produce structural clusters as a consequence of learning, there is not a guaranteed correspondence between the structural clusters and activation level in a complex task like image classification.

**Fig 5:**
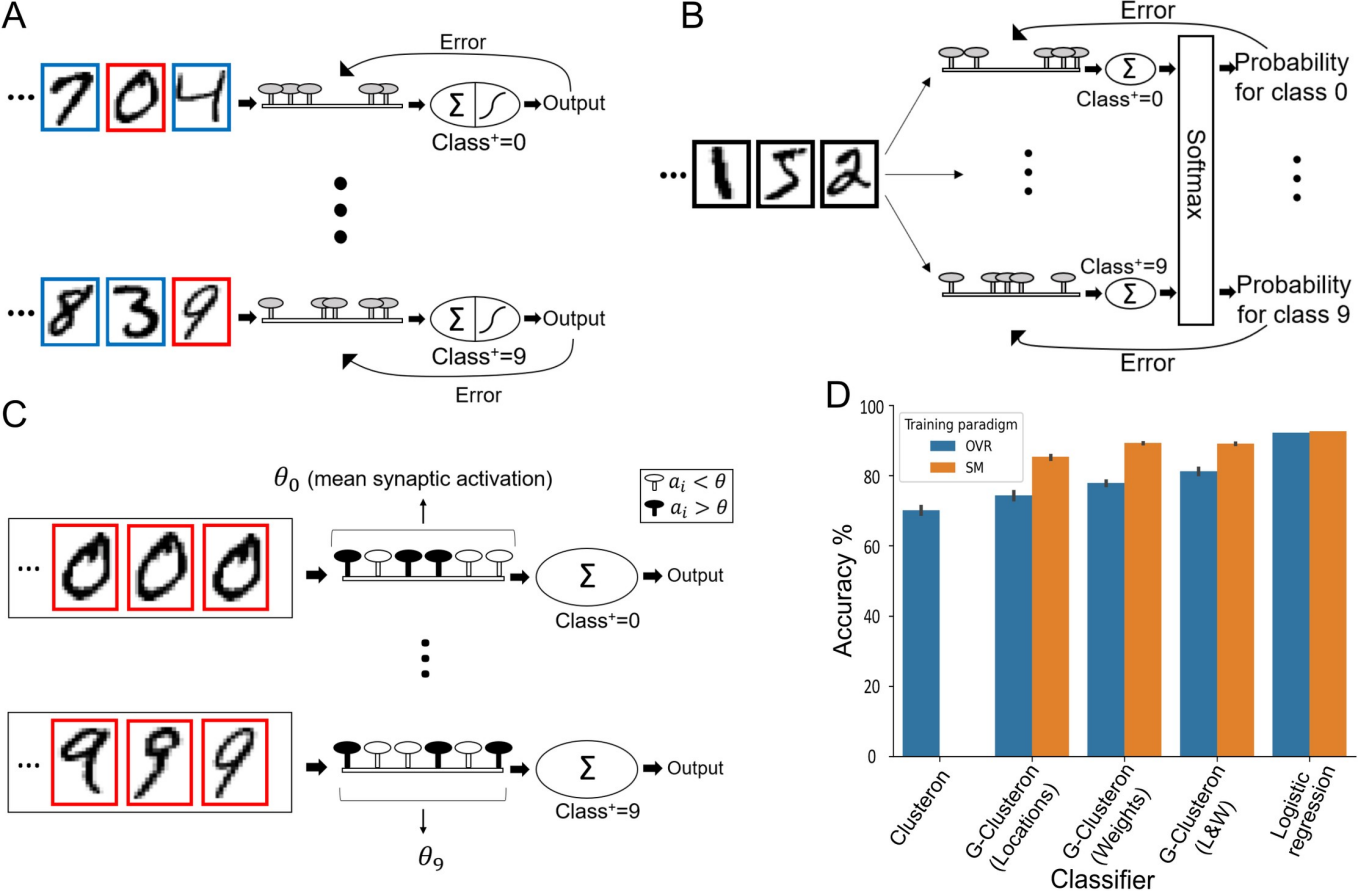
Multiclass classification with the G-clusteron on handwritten digits (MNIST). (A) One-versus-rest (OVR) learning scheme for the G-clusteron. Classifiers for each digit are trained independently on both positive (red border) and negative (blue border) digits. (B) Softmax (SM) classification scheme for the G-clusteron. Classifiers are trained simultaneously on each example and the sums of the synaptic activations for each classifier are fed into a softmax function which normalizes the output of each classifier by the output of all the classifiers. The error term used to update each classifier for an input digit thus has information about the output of all the classifiers for that digit. (C) OVR learning scheme for the original clusteron. On each epoch, each classifier is presented with an entire dataset consisting only of positive examples (red borders), and *θ*—the mean synaptic activation over the dataset—is calculated. The clusteron learns by randomly shuffling the locations of synapses whose activation was less than *θ*. Because each classifier only trains on positive examples and because there is no error term that can be communicated to other classifiers, the original clusteron does not lend itself to a softmax architecture as in B. (D) Mean accuracy for clusteron, G-clusteron (with either only the location update rule, only the weight update rule, or both rules), and logistic regression on the all-versus-all MNIST task using either the OVR method (blue) or SM method (orange). Error bars indicate standard deviation. Note that the original clusteron cannot use the SM method.

Having shown that the clusteron and G-clusteron exhibit satisfactory performance on the one-versus-all MNIST task, we now turn to the harder problem of all-versus-all classification. Here, we wish to train a single-layer network of 10 classifiers on the MNIST dataset (one for each digit) and have the network classify each digit correctly. We consider two standard ways to train a single-layer network on a multiclass classification problem. In the one-versus-rest (OVR) method, 10 units are independently trained on a one-versus-all paradigm, as before, and the classifier which produces the largest output ($\hat{y}$) for a given input pattern is declared the “winner”, and the input pattern is assigned to the positive class for that classifier (Fig 5A). In the softmax (SM) method, the units are trained simultaneously on each example and the raw outputs (*h***(*x*)**) of all units are passed through a softmax function (see **Methods**), which normalizes the output of each unit by the sum of the outputs of all the units in the layer (Fig 5B). This normalized output is then used in the error term $\hat{y}-y$ when calculating the update rule. Because the softmax method allows the classifiers to communicate with each other via the output normalization, it can often lead to superior results for multiclass classification [37].

Importantly, the original single-dendrite clusteron does not utilize an error signal in its learning rule, so the original clusteron can only be trained on the multiclass task with the OVR paradigm (Fig 5C). Both logistic regression and the G-clusteron, however, do use an error term, making it straightforward to perform multiclass classification with both the OVR and SM methods.

To test the performance of the G-clusteron under both multiclass classification paradigms, we trained the original clusteron, the G-clusteron, and logistic regression using the OVR method, and the G-clusteron and logistic regression (but not the original clusteron) using the SM method. As with the previous task, learning for all classifiers was repeated 10 times to ensure stability.

When using the OVR paradigm, the original clusteron achieved an average accuracy of 70.1%, the G-clusteron achieves 74.3% accuracy, and logistic regression achieves 92.2% accuracy. While both the original clusteron and the G-clusteron achieve performance far better than chance level of 10%, neither of them do nearly as well as logistic regression. However, when we train the G-clusteron using the SM method, it achieves an impressive accuracy of 85.3%, closing in on logistic regression’s 92.6% accuracy in the SM scheme (Fig 5D). Although not superior to logistic regression, 85.3% accuracy on an all-versus-all MNIST classification task is notable for an algorithm that can only modify synaptic locations and not synaptic weights.

### Synaptic weight update rule for the G-clusteron

In addition to the location update rule, we also derive a gradient descent rule for the weights of the G-clusteron of the form:

$$w_{i}\leftarrow w_{i}+\Delta w_{i}$$ (10)

Where Δ*w*_*i*_ is the update to the weight of synapse *i* defined as (assuming a logit or cross-entropy error loss function, see **Methods**):

$$\Delta w_{i}=-\eta_{W}\left(\hat{y}-y\right)\frac{a_{i}}{w_{i}}$$ (11)

with *η*_*W*_ as the positive-valued learning rate for the weights. This rule can either be used on its own or in conjunction with the location update rule (Eq 6) by simultaneously updating the weights and locations using their respective rules during each epoch. To see if the weight update rule can improve the accuracy of the G-clusteron on the all-versus-all MNIST task, we trained a single-layer G-clusteron network that used only the weight update rule as well as a network that used both the location update rule and the weight update rule simultaneously, using both the OVR and SM protocols.

When only the weight update rule was used, the G-clusteron achieved an accuracy of 77.9% for OVR and 89.3% for SM, and when both the weight and location update rules were used simultaneously, the G-clusteron achieved an improved accuracy of 81.2% for OVR and a similar accuracy of 89.1% for SM. Thus, being able to manipulate synaptic weights may allow the G-clusteron to achieve slightly superior accuracy relative to a G-clusteron that can only update its synaptic locations (Fig 5D).

Although the G-clusteron with both the weight and location update rules did not perform as well as logistic regression on the MNIST task, this does not indicate that the theoretical classification capacity of the G-clusteron is inferior to that of a linear classifier. In fact, we prove (see **S3 Text**) that if a G-clusteron with an arbitrary localization of synapses is equipped with a single additional “bias synapse” *x*_0_ (*x*_0_ = 1 for all input patterns, this is distinct from the 0^th^-order bias term *b*), it can approximate a linear classifier to an arbitrary degree of precision by appropriately assigning the weights. The discrepancy in classification accuracy in this task between logistic regression and the G-clusteron with a weight update rule is thus likely due to the G-clusteron’s failure to converge to a global minimum of the loss function rather than an inability to implement a linear separation boundary. Future optimizations to the learning algorithm might therefore allow the G-clusteron to surpass the accuracy of logistic regression on MNIST. In any event, we will now show that the G-clusteron is indeed superior to a linear classifier with respect to one particularly famous task: the XOR problem.

### XOR problem

To motivate the use of the weight update rule in combination with the location update rule for the G-clusteron, we consider the XOR function. The XOR function receives two bits of binary input (*x*_1_ and *x*_2_) and returns a 1 if the input bits are different or a 0 if the input bits are the same. The XOR function famously cannot be implemented by linear classifiers like the perceptron [34]. It is thus valuable to demonstrate that the G-clusteron can implement this function. We first wish to show that there are values for the parameters (i.e. the weights—*w*_1_ and *w*_2_—and the locations—*l*_1_ and *l*_2_—of the two synapses) of the G-clusteron that will result in a correct implementation of the XOR function. We then show that a G-clusteron must be able to update both its synaptic locations and its synaptic weights if it is to learn to solve the XOR problem from every arbitrary initialization of its parameter values.

To implement the XOR function, the parameters of the G-clusteron must satisfy both of the following inequalities (See **Methods**):

$$w_{2}^{2}<-2∙F_{12}∙w_{1}w_{2}$$ (12)

$$w_{1}^{2}<-2∙F_{12}∙w_{1}w_{2}$$ (13)

**Fig 6:**
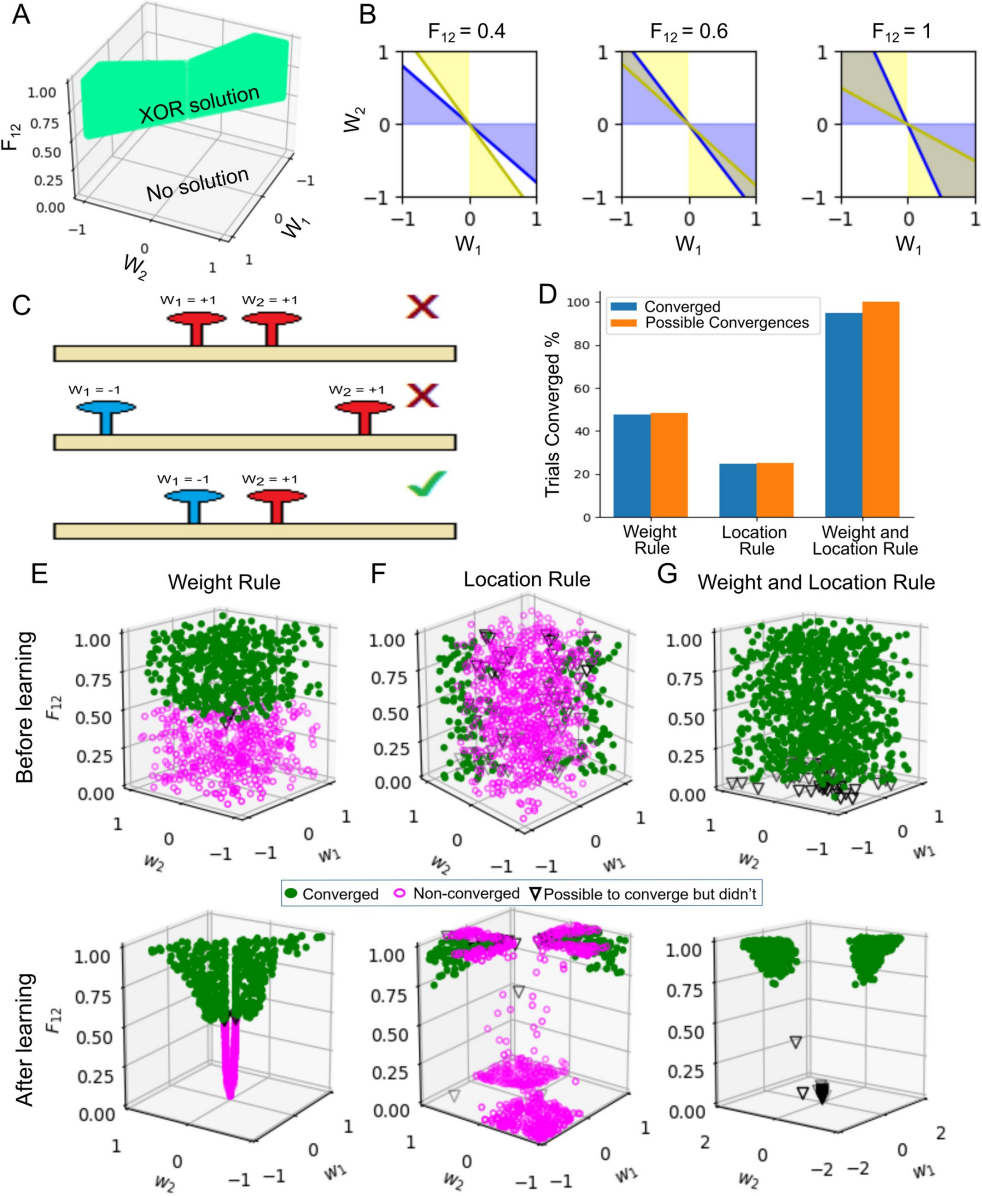
The G-clusteron requires the weight and location update rules to solve the XOR problem from arbitrary initial conditions. (A) Analytical solution to XOR problem. Green area represents the parameter space in terms of the two weights, *w*_1_ and *w*_2_, and the distance-dependent factor, *F*_12_, which would produce the correct outputs for the XOR problem. (B) Representation in weight space of the analytical solution to XOR, shown for selected values of *F*_12_ (i.e. slices in the *F*_12_ axis of A). The blue region satisfies the inequality from Eq 12, and the yellow region satisfies the inequality from Eq 13. The G-clusteron can solve the XOR problem when both inequalities are satisfied (overlap region, same as green region in A). Note that the solution space for the weights is null when *F*_12_<0.5 and increases as *F*_12_ approaches 1. However, there are many weight assignments that never satisfy both inequalities. (C) Examples of correct and incorrect parameter assignments for the XOR problem. Some weight assignments never produce a correct solution for XOR regardless of the distance between the synapses (top). Moreover, if the synapses are too far away from each other, there are no weights that satisfy the XOR relation (middle). Thus, solving XOR from arbitrary initial conditions requires that the synapses be close together and the weights appropriately set (bottom). (D) Number of trials that were analytically possible to converge versus trials that successfully converged on the XOR problem for the G-clusteron trained with only the weight update rule, only the location update rule, or both rules. (E) Top: Convergence (marker shape and color, see legend) as a function of the initial parameters of 1000 randomly initialized G-clusterons with only the weight update rule. Note that only trials in which the initial value for *F*_12_ was greater than 0.5 converged. Bottom: Convergence as a function of the final parameters of the G-clusterons after learning. Note that converged trials fall within the region specified in A. (F) Same as E for G-clusteron using only the location update rule. Note that only trials in which the initial values for the weights were within the overlap region of B (right panel) were able to converge. (G) Same as E for G-clusteron with both the weight and location update rules. Almost all trials converge to the correct solution.

Note that although we originally have 4 parameters (*w*_1_, *w*_2_, *l*_1_, and *l*_2_), the solution space satisfying these inequalities (Fig 6A and 6B) can be expressed in terms of only three parameters: *w*_1_, *w*_2_, and *F*_12_.

When expressed in this manner, there are several things to observe about these inequalities. First, note that for *F*_12_≤0.5, there is no way to assign the weights such that Eqs 12 and 13 are satisfied (Fig 6B, left). This means that if the two synapses are initialized sufficiently far away from each other such that their distance-dependent factor is less than 0.5, a G-clusteron with only a weight update rule (Eq 10) would have no way to solve the XOR problem.

Moreover, the range of weights that are valid to solve the XOR problem increases as *F*_12_ increases from 0.5 to 1, where the solution space for the weights is maximal (Fig 6B, center and right). In other words, as the synapses move closer together, there is a larger set of weights that would satisfy Eqs 12 and 13. However, even when the synapses occupy the exact same location (i.e. *F*_12_ = 1), there is still a large range of invalid weights. For example, if both weights are initialized with the same sign, a G-clusteron with only a location update rule (Eq 6) could not implement the XOR function (Fig 6B and 6C). Therefore, for the G-clusteron to solve XOR from arbitrary initial parameter values, it must be able to update both its weights and its locations.

To demonstrate this numerically, we created 3 G-clusterons: one with only a weight update rule, one with only a location update rule, and one with both update rules. For each G-clusteron, we ran 1000 trials with randomized initializations of the inputs and labels for the XOR problem. Each trial ran for 10,000 epochs or until convergence (see **Methods**).

For a G-clusteron with only the weight update rule, a trial was designated as “possible to converge” if some weight assignment could produce the correct XOR output given the initialization of the two synaptic locations, which can only occur if the initial *F*_12_ was greater than 0.5. For a G-clusteron with only the location update rule, a trial was designated as “possible to converge” if some location assignment could produce the correct XOR output given the initialization of its synaptic weights. Because the solution space for XOR grows as the synapses move closer to each other, this entails that the initial weights would have to satisfy Eqs 12 and 13 given that *F*_12_ = 1 (in other words, the weights must fall in the overlap shaded region of Fig 6B, right panel). For the G-clusteron with both the weight and the location update rules, all trials could potentially converge (Fig 6D).

The G-clusteron with only the weight update rule converged for 475 trials out of 485 trials analytically possible to converge (Fig 6D and 6E), unexpectedly failing on trials where *F*_12_ was initialized slightly above the boundary condition of 0.5, because the solution space for the weights when *F*_12_ is fixed near 0.5 is small (Fig 6B middle), and it can therefore take the learning algorithm an exorbitantly long time to direct the weights into this exactingly narrow range of values.

The G-clusteron with only the location update rule converged for 247 trials out of 251 trials analytically possible to converge (Fig 6D and 6F), unexpectedly failing on trials where *F*_12_ was initialized very close to 0, as before, and also on trials where the weights were initialized very close to the boundary of the solution space (Fig 6B, right panel). In these cases, it was evidently difficult for the algorithm to find the bias value with sufficient precision to correctly perform the classification, even though the weights and locations were analytically valid.

Only the G-clusteron with both the weight and the location update rules was able to converge on almost all of the trials (947 out of 1000 trials), unexpectedly failing only on trials where *F*_12_ was initialized very close to 0 (Fig 6D and 6G). These unexpected failures occurred because the magnitude of the location update rule for a given epoch scales with the value of *F*_12_ and the weights at that epoch (Eq 7). Thus, if *F*_12_ is initialized very close to 0, the synaptic locations may only move a miniscule amount each time the gradient rule is applied, depending on the values of the other parameters.

## Discussion

We have shown that the G-clusteron is a robust single-neuron learning algorithm that can solve real-world classification tasks, such as MNIST handwritten digit classification. The G-clusteron’s ability to solve this task merely by moving synapses on its dendrite without using synaptic weights demonstrates the computational potential of structural plasticity that makes use of distance-dependent nonlinearities.

While the original single-dendrite clusteron [28] also exhibits impressive performance when tasked with one-versus-all classification on a single digit, the lack of a supervised gradient descent-based plasticity rule makes it difficult to scale this algorithm to a multiclass classification problem, as the clusteron learning rule does not have an error signal that can be communicated between neurons within a layer via a softmax activation function.

The gradient descent plasticity rule of the G-clusteron enables effective multiclass classification and allows the use of a variety of techniques developed for classic ANNs. For example, we used the ADAM momentum-based adaptive learning method [38] to dynamically optimize our learning rate. Bringing dendritic cluster-based plasticity algorithms within the fold of gradient descent learning opens up many possibilities for the extension of the G-clusteron algorithm using the extant mathematical frameworks and robust literature for learning with gradient descent in ANNs [39].

We have also shown that incorporating a plasticity rule for the weights in a G-clusteron alongside the dendritic location plasticity rule enables the G-clusteron to solve the classic linearly inseparable XOR problem [34]. This reinforces the possibility that neuron models with dendritic nonlinearities may be more computationally powerful than the linear nodes used in ANNs [40–43].

### Relationship to previous work

The original clusteron model [28] has been developed into the “two-layer model” [40, 41] in which synapses are linearly summed within dendritic branches, and each branch computes a sigmoidal nonlinearity of its input before sending its output to the soma where a final nonlinearity is applied. Unlike the original clusteron, this two-layer model does employ a supervised learning strategy that applies gradient descent to modify synaptic stability, which in turn determines how synapses are mapped to individual branches [40], however it does not directly learn exact synaptic locations via gradient descent as we do here. The two-layer model has been shown to achieve above 96% accuracy on a binary MNIST classification task (although not directly comparable to the tasks in our study, this result was superior to a linear classifier) [44]. A two-layer neuron with two branches can also implement the XOR function by, for example, having one branch implement OR, the other branch implement NAND, and the soma implement AND, each of which is a linear operation. We emphasize that the G-clusteron, as a single-branch model, is not in competition with the two-layer model in terms of classification performance. Rather, the G-clusteron is a model of very local, distance-dependent nonlinearities that may occur within a single dendritic branch. The sigmoidal output of the G-clusteron can be interpreted as a branch-level nonlinearity (as opposed to a somatic nonlinearity) and the G-clusteron can thus be incorporated as the branch-level unit in a two-layer model. This would provide an interesting avenue of future research.

In addition to the two-layer model [40, 41], there have been a variety of other attempts to model learning with dendrites and dendritic nonlinearities [45–47]. These models also tend to treat the dendritic branch (or sometimes an entire dendritic tree) as discrete loci of nonlinearity. Conceivably, both discrete, branch- or tree-dependent nonlinearities and fluid, location-dependent nonlinearities could exist simultaneously, further expanding the computational capabilities of real neurons.

Although our work here treats dendrites with NMDA receptors as nonlinear integration units, recent work [48] has shown that a detailed biophysical model of a cortical layer 5 pyramidal cell with NMDA synapses and other active mechanisms can implement the perceptron learning algorithm, implying that a neuron can behave as a linear classifier. However, this does not preclude the possibility of nonlinear integration and plasticity rules; rather it should be taken as an indication a neuron can elect to apply a simple plasticity rule that ignores the synergistic interactions between synapses and still manage to solve classification tasks as a perceptron would. Moreover, Mel [49] has shown that a clusteron-like algorithm can also be implemented in a detailed biophysical model of a cortical pyramidal cell with NMDA synapses.

The original clusteron [28] fits within the framework of subsampled quadratic classifiers, as the clusteron sums a subset of its *N*^2^ mixed terms *x*_*i*_*x*_*j*_ [42]. The G-clusteron can be thought of as a “constrained” quadratic classifier in the sense that the equation for the G-clusteron contains all *N*^2^ mixed terms, but the coefficients of these terms–the entries of the *F* matrix–are “tied together” in the sense that *F* must be produced from a matrix representing distances between points in one dimension, and not all matrices are valid distance matrices. The G-clusteron therefore gives us *N*^2^ mixed terms for the price of *N* location parameters, but at the cost of not being able to manipulate each of the *N*^2^ coefficients independently.

From a computational standpoint, we note that the covariance perceptron [50] also takes a correlation-based approach to solving learning tasks.

### Biological plausibility

The G-clusteron takes several liberties with respect to the biological phenomena from which it is inspired. While a multiplicative activation function may be a tolerable first-order approximation of the synergetic cooperation between NMDA synapses, a sigmoidal function provides a closer fit [41]. Even to the extent that a multiplicative model does portray the synergistic relationship between excitatory NMDA synapses, such a relationship does not exist between excitatory synapses and inhibitory synapses or between inhibitory synapses and other inhibitory synapses. The multiplicative interaction with negative inputs, however, is essential to the learning protocol of the G-clusteron, so this aspect of our algorithm should be viewed as “biologically inspired” rather than an accurate depiction of what occurs in real biological cells. We do note, however, that clustering dynamics have been observed to occur between dendritic spines and inhibitory synapses [51].

Although in real biological neurons there is voltage attenuation from the dendrite to the soma [52, 53], we have chosen not to include this feature in the G-clusteron in order to emphasize the effect of local interactions, to maintain similarity to the original clusteron model [28], and to keep the model relatively simple. This choice can be justified biologically either on the basis of “synaptic democracy” [54, 55], where synaptic weights are adjusted to compensate for attenuation, or by noting that in some cases the high input resistance of distal dendritic regions may help balance out attenuation to the soma in active dendrites [56].

While some have posited that the dendritic branch may be the fundamental computational unit within a neuron [57, 58], there is reason to believe that interesting clustering dynamics occur within branches on a more local scale. Although there is no consensus definition for synaptic clustering, several studies have shown that cluster-related dynamics occur on a scale from 0–15 μm. Synapses are more likely to exhibit correlated activity if they are within 10 μm of each other [14]. Relatedly, the intercontact distance (ICD) between synapses decreases from ~10 μm to ~5 μm in owl inferior colliculus following an adaptation protocol [19]. Moreover, the relevant range for BDNF-dependent clustering seems to be ~15 μm [11], and the spread of Ras, which allows “crosstalk” between synapses in a long-term potentiation (LTP) protocol, is limited to ~10 μm [59, 60]. Heterosynaptic plasticity—structural long-term depression (LTD) occurring near spines that underwent structural LTP—also occurs on a scale of ~10 μm [21].

The mechanism of learning synaptic locations in the G-clusteron, namely the attraction and repulsion of synapses based on the activity of their presynaptic inputs, also warrants discussion from a biological standpoint. As we have described, the computation of the “forces” exerted by each synapse results in a vector field along the dendrite with regions of attraction and repulsion. Such a vector field could be implemented by the release of attractive or repulsive chemical factors in proportion to the local dendritic activation which diffuse in the extracellular space, creating a distance-dependent gradient that effectively sums the “pull” of the synapses that were active along the dendrite. The attractive factors could stimulate the growth of presynaptic axonal boutons or postsynaptic filopodia at specific regions of the dendrite, while the repulsive factors could eliminate existing synapses.

Brain-derived neurotrophic factor (BDNF) and its precursor, proBDNF, are strong candidates for the signaling mechanism underlying the sort of structural plasticity we suggest here. BDNF has been shown to be responsible for structural plasticity in development by stabilizing correlated synapses during development, while proBDNF weakens synapses that exhibit uncorrelated activity [11]. Another possible signaling agent is estradiol, which is also involved in the formation of new spines [61] and interacts with BDNF in spine regulation pathways [62]. Astrocytes and microglia, which play an important role in spine elimination [63] may also be implicated in our model as a mechanism for intelligently rearranging synapses on the dendrite in response to local activity.

We note that the NMDA receptor is not only a mechanism for voltage nonlinearities; the receptor also plays a crucial role in the plasticity process itself by allowing Ca^2+^ ions to enter into the postsynaptic cell, initializing a cascade that can result in LTP or LTD, depending on the stimulation protocol used [64, 65]. Interestingly, the NMDA receptor is involved in structural plasticity as well, as the release of proBDNF and BDNF is NMDA-dependent [11, 12]. Moreover, there is evidence that both LTP and LTD protocols can cause structural plasticity changes, with LTP encouraging the growth of new spines and LTD resulting in spine retraction [66]. This lends support to the idea that structural and functional plasticity may be happening simultaneously, both mediated by the NMDA receptor.

Several testable experimental predictions emerge from the G-clusteron’s location update rule. If presynaptic axons are indeed being coaxed into moving their synapses along chemical gradients, we might expect to see axonal boutons being destroyed and new boutons from the same axon forming a short distance away. We may also expect to see different clustering patterns on different regions of the dendrite due to different lengths constants, as in Fig 2A and 2B. Because the length constant *λ* increases with dendritic diameter [35, 53], we might expect that thinner regions of dendrite would have a larger number of small clusters than wider regions, which may tend to group synapses into a smaller number of large clusters. However, these effects would likely depend on the exact nature of the task that the neuron performs.

The distance-dependence of the weight update rule (Eq 11) of the G-clusteron can also be understood in a biological framework. Experimental evidence has shown that when LTP is induced in one synaptic spine, the threshold for LTP induction in nearby spines within ~10μm is reduced [67], a process mediated by the intracellular diffusion of Ras [59]. The G-clusteron’s weight update rule qualitatively expresses a similar phenomenon–if a synapse has a strong input such that its weight increases a large amount, nearby synapses will require less input to achieve a large weight update.

Both the weight and location update rules in our model require an error signal ($\hat{y}-y$). A prominent candidate for this error signal is the neuromodulator dopamine, which has been shown to correspond to reward prediction error [68]. Dopamine has also been demonstrated to be necessary for LTP in hippocampal CA1 [69] and is involved in learning and plasticity processes in cortex [70], adding support to the idea that dopamine-mediated error signals play an important role in supervised learning.

### Future directions

The G-clusteron can be extended in a variety of directions. Because the G-clusteron is inspired by biological dendrites, we use one-dimensional synaptic locations, but our model and algorithm can be modified such that synapses interact with each other as a function of distance in a space with arbitrarily high dimensions, instead of merely moving along a line. Presynaptic axons ostensibly do not just need to localize their boutons within a branch; they may also want to decide between branches of the same neuron or among different neurons. As such, a 2D or 3D G-clusteron, where synapses are localized within the plane of a neuron’s branching structure or within a 3D volume of brain tissue, also has some biological motivation. The 2D or 3D case may be more relevant during early development, when the brain is being wired up for the first time, whereas the one-dimensional case presented here may be more relevant to later stages of development or adulthood, when the brain is densely packed and neuronal processes are likely more restricted in their motion, possibly limited to resampling locations along a dendritic branch.

Our model can also be made more biologically plausible in a number of ways, such as by using a sigmoidal nonlinearity instead of a multiplicative nonlinearity [40, 41], incorporating recent work regarding distance-dependent interactions between nonlinear synapses [71–73], or incorporating attenuation from the dendrite to the soma [52, 53]. One recent study [74] used an attenuation-based model to show how neurons could learn probability distributions via creating multiple synaptic connections at different dendritic locations. It would be valuable to see how incorporating these features into the G-clusteron model would affect both the weight and location gradient update rules.

As with any single-neuron model, the G-clusteron should also be explored in the context of a multi-layer network. The G-clusteron’s gradient descent-based update rules lend themselves to the possibility of a backpropagation algorithm for a deep network of G-clusteron neurons. This can create exciting directions for a version of deep learning that incorporates synaptic weight updates together with dendritic nonlinearities and structural plasticity.

## Methods

### Derivation of location update rule for the G-clusteron

We wish to derive a learning rule that updates the synaptic locations on each iteration of the algorithm of the form:

$$l_{i}\leftarrow l_{i}+\Delta l_{i}$$

Where Δ*l*_*i*_ is proportional to the gradient of the error with respect to the locations. For an arbitrary loss function *J* and nonlinearity *g* where we have *J*(*g*(*h*(*l*_*i*_))) we have:

$$\Delta l_{i}=-\eta_{L}\frac{\delta J}{\delta l_{i}}$$

By the chain rule we have:

$$\frac{\delta J}{\delta l_{i}}=\frac{\delta J}{\delta g}\frac{\delta g}{\delta h}\frac{\delta h}{\delta l_{i}}$$

The first two factors of the gradient, $\frac{\delta J}{\delta g}\;and\;\frac{\delta g}{\delta h}$, are specific to the cost function and non-linearity chosen. In our case, we use the logit cross-entropy error

$$J(\theta )=-y\log(\hat{y})-(1-y)\log(1-\hat{y})$$ (14)

and sigmoidal nonlinearity

$$g(h)=\frac{1}{1+e^{-h}}$$ (15)

We therefore have (see [75] for elaboration):

$$\frac{\delta J}{\delta g}\frac{\delta g}{\delta h}=\hat{y}-y$$ (16)

For the final term (see **S4 Text** for full derivation of $\frac{\delta h}{\delta l_{i}}$):

$$\frac{\delta h}{\delta l_{i}}=\frac{4}{r}\sum_{j=1}^{N}(l_{j}-l_{i})F_{ij}w_{i}x_{i}w_{j}x_{j}$$

We thus have:

$$\frac{\delta J}{\delta l_{i}}=(\hat{y}-y)\frac{4}{r}\sum_{j=1}^{N}{(l_{j}-l_{i})F}_{ij}w_{i}x_{i}w_{j}x_{j}$$

We can subsume the constant factor $\frac{4}{r}$ into the learning rate *η*_*L*_, which gives:

$$\Delta l_{i}=-\eta_{L}(\hat{y}-y)\sum_{j=1}^{N}(l_{j}-l_{i})F_{ij}w_{i}x_{i}w_{j}x_{j}$$

Which gives our location update rule (Eq 7). This same expression holds if we use a softmax nonlinearity:

$${\hat{y}}_{i}=\frac{e^{h_{i}}}{\sum_{j}e^{h_{j}}}$$

and a cross-entropy cost function:

$$J(\theta )=-\sum_{i}y_{i}log({\hat{y}}_{i})$$

as we do for the multiclass MNIST classification task (See [75]). (Here *i* and *j* refer to the class labels in a multiclass classification task).

### Derivation of bias update rule

We also require a rule for the update of the bias term *b*. As above:

b←b+Δb (17)

$$\Delta b=-\eta_{B}\frac{\delta J}{\delta b}$$

And

$$\frac{\delta J}{\delta b}=\frac{\delta J}{\delta g}\frac{\delta g}{\delta h}\frac{\delta h}{\delta b}$$

The first two terms are as above, the final term is:

$$\frac{\delta h}{\delta b}=-1$$

so we have

$$\frac{\delta J}{\delta b}=-(\hat{y}-y)$$

And

$$b=\eta_{B}(\hat{y}-y)$$ (18)

Where *η*_*B*_ is the positive-valued bias learning rate. (Note the difference in sign relative to the location and weight update rules.)

### Derivation of weight update rule

In addition to the location update rule, we also derive a gradient descent rule for the weights of the G-clusteron of the form:

$$w_{i}\leftarrow w_{i}+\Delta w_{i}$$

Where:

$$\Delta w_{i}=-\eta_{W}\frac{\delta J}{\delta w_{i}}$$

We have

$$\frac{\delta J}{\delta w_{i}}=\frac{\delta J}{\delta g}\frac{\delta g}{\delta h}\frac{\delta h}{\delta w_{i}}$$

As with the location update rule, we have:

$$\frac{\delta J}{\delta g}\frac{\delta g}{\delta h}=\hat{y}-y$$

Taking the derivative $\frac{\delta h}{\delta w_{i}}$ we obtain (see **S5 Text** for full derivation of $\frac{\delta h}{\delta w_{i}}$):

$$\frac{\delta h}{\delta w_{i}}=2x_{i}\sum_{j=1}^{N}F_{ij}w_{j}x_{j}=\frac{2a_{i}}{w_{i}}$$

We can subsume the constant factor 2 into the learning rate. The weight update rule for the G-clusteron is thus:

$$\Delta w_{i}=-\eta_{W}(\hat{y}-y)\frac{a_{i}}{w_{i}}$$

### Efficient computation and batch learning

For computational efficiency on the MNIST task, the output of the G-clusteron and the learning rules can be implemented in a vectorized fashion. We assume here that we will be working with a dataset comprised of multiple input patterns, with indices *p*∈1,2…*P*. We will denote input *j* in pattern *p* as $x_{j}^{\left(p\right)}$. To efficiently calculate each synaptic activation over an entire dataset, we define a matrix *S* such that

$$S_{pj}=w_{j}x_{j}^{\left(p\right)}$$ (19)

We also define a signed distance matrix *D* where:

$$D_{ij}=l_{j}-l_{i}$$ (20)

The distance-dependent factor matrix *F* can be expressed in terms of *D*:

$$F_{ij}=e^{\frac{-D_{ij}^{2}}{r}}$$ (21)

The activation for synapse *j* in pattern *p*, $a_{j}^{\left(p\right)}$, is the element *A*_*pj*_ of matrix *A*, which can be computed as:

A=S°(SF) (22)

Where ° denotes the Hadamard product, i.e. elementwise multiplication.

For the location update rule, we utilize a batch protocol where updates are performed after observing *P* patterns (*P* here is the size of the batch, not the dataset). Here, for each pattern *p* in the batch we define a matrix *Q*^(*p*)^ such that

$$Q_{ij}^{\left(p\right)}=w_{i}x_{i}^{\left(p\right)}w_{j}x_{j}^{\left(p\right)}$$ (23)

The derivative of the loss function on the entire batch with respect to location *l*_*i*_ is given by:

$$\frac{\delta J}{\delta l_{i}}=\frac{1}{P}\sum_{p=1}^{P}\frac{\delta J^{\left(p\right)}}{\delta l_{i}}=\frac{1}{P}\sum_{p=1}^{P}({\hat{y}}^{\left(p\right)}-y^{\left(p\right)})\frac{4}{r}\sum_{j=1}^{N}(l_{j}-l_{i})f(l_{i},l_{j})w_{i}x_{i}^{\left(p\right)}w_{j}x_{j}^{\left(p\right)}$$ (24)

Using our matrix notation, we have

$$\frac{\delta J}{\delta l_{i}}=\frac{1}{P}\sum_{p=1}^{P}({\hat{y}}^{\left(p\right)}-y^{\left(p\right)})\frac{4}{r}\sum_{j=1}^{N}{\left[F{}^{\circ}D{}^{\circ}Q^{\left(p\right)}\right]}_{ij}$$ (25)

However, this is computationally intensive as it requires an elementwise multiplication for every input pattern in the batch (*F*°*D* can be precomputed for the entire batch, but *F*°*D*°*Q*^(*p*)^ is different for every input pattern). We therefore rearrange and obtain:

$$\frac{\delta J}{\delta l_{i}}=\frac{4}{r}{\sum_{j=1}^{N}\left[F^{\circ }D^{\circ }(\frac{1}{p}\sum_{p=1}^{P}\left({\hat{y}}^{\left(p\right)}-y^{\left(p\right)}\right)Q^{\left(p\right)})\right]}_{ij}$$ (26)

Which averages over all input patterns in the batch first, requiring only two elementwise multiplications regardless of batch size.

For the weight update rule we have:

$$\frac{\delta J}{\delta w_{i}}=\frac{1}{P}\sum_{p=1}^{P}\frac{\delta J^{\left(p\right)}}{\delta w_{i}}=\frac{1}{P}\sum_{p=1}^{P}({\hat{y}}^{\left(p\right)}-y^{\left(p\right)})\frac{{2a_{i}}^{\left(p\right)}}{w_{i}}=\frac{2}{P}\sum_{p=1}^{P}({\hat{y}}^{\left(p\right)}-y^{\left(p\right)})\frac{A_{pi}}{w_{i}}$$

Which can be efficiently computed for all weights in all patterns simultaneously by multiplying each row *p* of *A* by ${\hat{y}}^{\left(p\right)}-y^{\left(p\right)}$ and dividing each column *i* of *A* by *w*_*i*_.

### Toy examples

To train the G-clusteron to continually increase or decrease its activation on a particular input pattern or dataset, we treated the activation function as a threshold function which always returned the wrong answer such that it would perform the maximal update at each epoch. For patterns with label *y* = 0, we fixed $\hat{y}=1$ such that $\hat{y}-y=1$, for patterns with label *y* = 1, we fixed $\hat{y}=0$ such that $\hat{y}-y=-1$.

### Learning MNIST

For the MNIST learning tasks, we used the Tensorflow [76] MNIST dataset [36], which is composed of 60,000 training examples and 10,000 test examples. The examples are split roughly evenly between the 10 digits. As a preprocessing step, we centered the data such that each image had a mean pixel value of 0.

The logistic regression implementation used as our baseline for comparison on MNIST was the default implementation in the Scikit-learn library [77], which uses the ‘LBFGS’ solver [78] and an L2 regularization penalty. Using the stochastic average gradient (‘SAG’) [79] solver and no regularization penalty achieves similar results (see repository linked in **Code and Data Availability Statement**). The original clusteron algorithm was implemented in Python by the authors using the description from [28].

For the one-versus-all experiments, the original clusteron was trained only on positive training examples, ~6,000 examples per digit. The G-clusteron and logistic regression classifiers were trained on a balanced dataset with ~6000 images of the digit from the positive class and ~6000 total images of other digits. The test set was evenly split between positive and negative examples. Hyperparameters and runtimes for the one-versus-all task can be found in Table 2.

**Table 2:** Hyperparameters for one-versus-all learning protocols.

	Epochs	Batch size	*η*_*B*_	*η*_*W*_	*η*_*L*_	Runtime (seconds)
**Clusteron**	100	~	~	~	~	810
**G-clusteron**	100	50	0.04	0	5*10^−5^	700
**Logistic regression**	Default logistic regression algorithm from the Scikit-learn library [77]. Parameters: {solver: ‘lbfgs’, max_iter: 100, multiclass: ‘ovr’}					OVR: 67 SM: 23

For the all-versus-all experiments, each of the 10 clusteron units were trained on all images from their positive class. Each G-clusteron and logistic regression classifier was trained with the entire MNIST dataset. Hyperparameters and runtimes for all-versus-all tasks are shown in Table 3.

**Table 3:** Hyperparameters for all-versus-all learning protocols. Abbreviations: WR/LR/BR: weight/location/both rules, respectively. OVR: one-versus-rest, SM: softmax.

	Epochs	Batch size	*η*_*B*_	*η*_*W*_	*η*_*L*_	Runtime (seconds)
**Clusteron**	100	~	~	~	~	827
**G-clusteron (WR, OVR)**	100	100	0.04	10^−4^	0	395
**G-clusteron (LR, OVR)**	100	100	0.04	0	4*10^−5^	1986
**G-clusteron (BR, OVR)**	100	100	0.04	10^−4^	4*10^−5^	1095
**G-clusteron (WR, SM)**	2000	30	10^−5^	10^−5^	0	317
**G-clusteron (LR, SM)**	2000	3	5*10^−6^	0	5*10^−6^	755
**G-clusteron (BR, SM)**	2000	5	10^−5^	10^−5^	10^−5^	961
**Logistic regression**	Default logistic regression from the Scikit-learn library [77]. Parameters: {solver: ‘lbfgs’, max_iter: 100, multiclass: ‘ovr’ for OVR or ‘multinomial’ for SM}					OVR: 67 SM: 23

For both one-versus-all and all-versus-all (SM and OVR), the hyperparameter *r* of the G-clusteron was always set to 0.23. For the original clusteron, synapses interacted if they were within 10 synapses of each other. Hyperparameters were hand-tuned to maximize accuracy in all cases.

### XOR problem

The XOR function is defined as (Table 4):

**Table 4:** XOR function.

Input		Output
*x*_1_	*x*_2_	*x*_1_ XOR *x*_2_
0	0	0
1	0	1
0	1	1
1	1	0

For the G-clusteron to solve the XOR problem, we require:

max(h(0,0),h(1,1))<min(h(0,1),h(1,0)) (27)

where *h*(***x***) is the output of the G-clusteron on the input vector ***x*** (In our case, the vector [*x*_1_, *x*_2_]) before applying the sigmoidal nonlinearity, as defined in Eq 4.

In the case of two inputs, *h*(***x***) can be written as:

$$h(x_{1},x_{2})=w_{1}x_{1}(w_{1}x_{1}+F_{12}w_{2}x_{2})+w_{2}x_{2}(F_{12}w_{1}x_{1}+w_{2}x_{2})-b$$ (28)

We therefore have (Table 5):

**Table 5:** G-clusteron outputs for two binary inputs.

Input		Output
*x*_1_	*x*_2_	*h*(*x*_1_, *x*_2_)
0	0	−b
1	0	$w_{1}^{2}-b$
0	1	$w_{2}^{2}-b$
1	1	$w_{1}^{2}+w_{2}^{2}+2∙F_{12}∙w_{1}w_{2}-b$

From Eqs 27 and 28 and Table 5, we require:

$$w_{1}^{2}+w_{2}^{2}+2\cdot F_{12}\cdot w_{1}w_{2}-b<w_{1}^{2}-b$$ (29)

and

$$w_{1}^{2}+w_{2}^{2}+2\cdot F_{12}\cdot w_{1}w_{2}-b<w_{2}^{2}-b$$ (30)

which can be simplified to Eqs 12 and 13 in **Results**. (Note that there are two other inequalities that follow from Eqs 27 and 28, namely that $w_{1}^{2}>0$ and $w_{2}^{2}>0$, which require that neither *w*_1_ nor *w*_2_ are equal to 0, however Eqs 29 and 30 already guarantee this.)

To test the different G-clusteron learning rules on the XOR dataset, we created a G-clusteron for each of the three learning paradigms: weight update rule only, location update rule only, and both weight and location update rules together. Each G-clusteron was run on 1000 trials with different parameter initializations for 10,000 epochs per trial.

For each trial, the weight values were chosen randomly from a uniform distribution within [-1,1], and the initial locations were chosen such that *F*_12_ was uniformly distributed within [0,1]. The G-clusterons were trained with a stochastic gradient descent protocol such that each epoch, one out of the four input vectors for the XOR function (Table 4) were presented to the G-clusteron, which would update its parameters according to the relevant update rule(s). For computational efficiency, we stopped the learning protocol on a given trial when the algorithm converged. Convergence was defined as achieving perfect accuracy on a test set of all 4 input vectors of the XOR problem for 10 consecutive epochs. Hyperparameters and the net runtime for all 10,000 trials are shown in Table 6.

**Table 6:** Hyperparameters for XOR problem.

XOR	*η*_*B*_	*η*_*W*_	*η*_*L*_	Net runtime (seconds)
Weight rule	0.0025	0.09	0	~8100
Location rule	0.0025	0	0.05	~11,880
Both rules	0.1	0.08	0.12	~864

For the XOR problem, the *r* hyperparameter was set to 1. Momentum was not used for the XOR problem. Hyperparameters were hand-tuned to maximize the number of converged trials. Runtimes differed due to different convergence probabilities for the different rules.

All scripts were written in Python and run on an Acer Aspire 5 Notebook laptop with an i7-10510U quad-core processor (1.80 GHz) and 16GB RAM, running on a Windows 10 operating system.

## Supporting information

S1 Movie (Movie for Fig 2A) Dynamics of synaptic clustering due to the G-clusteron location update rule, using a large value for the *r* parameter and trained on a single positive-labeled pattern. Synapse locations are initiated randomly, and then separated into groups of synapses with positive or negative inputs.
(MP4)

S2 Movie (Movie for Fig 2B) Same as S1 Movie but using a small value for the *r* parameter. (MP4)

S3 Movie (Movie for Fig 2C) Similar to S1 Movie, but trained on a negative example, with synapse locations initiated such that synapses are clustered with same-sign input values. Over the course of learning, clusters are dispersed, leading to lower overall activation.
(MP4)

S4 Movie (Movie for Fig 3C) Dynamics of synaptic clustering for a G-clusteron trained on positive-labeled input patterns from a multivariate Gaussian dataset with multiple groups of positively correlated inputs. (MP4)

S5 Movie (Movie for Fig 3D) Same as S4 Movie, but with negative-labeled input patterns. Clusters of negatively correlated inputs are formed over the course of learning.
(MP4)

S1 Text Explanation of signs and directionality in the location update rule. (PDF)

S2 Text Results for multiple trial runs of the multivariate Gaussian task. (PDF)

S3 Text Proof that the G-clusteron can approximate a linear classifier by adding an additional “bias synapse” and setting the weights and bias appropriately. (PDF)

S4 Text Extended derivation of location update rule. (PDF)

S5 Text Extended derivation of weight update rule. (PDF)

## References

[1] Lecun Y, Bengio Y, Hinton G. Deep learning. Nature. Nature Publishing Group; 2015. pp. 436–444. doi: 10.1038/nature14539 26017442

[2] Amit DJ. Modeling brain function: The world of attractor neural networks. Cambridge university press; 1992.

[3] Memmesheimer RM, Rubin R, Ölveczky BP, Sompolinsky H. Learning Precisely Timed Spikes. Neuron. 2014;82: 925–938. doi: 10.1016/j.neuron.2014.03.026 24768299

[4] Kriegeskorte N. Deep Neural Networks: A New Framework for Modeling Biological Vision and Brain Information Processing. Annu Rev Vis Sci. 2015;1: 417–446. doi: 10.1146/annurev-vision-082114-035447 28532370

[5] Rumelhart D, McClelland J, Group CPR. Parallel distributed processing: explorations in the microstructure of cognition, vol. 2: psychological and biological models. 1986.

[6] Saxe AM, McClelland JL, Ganguli S. A mathematical theory of semantic development in deep neural networks. Proc Natl Acad Sci U S A. 2019;166: 11537–11546. doi: 10.1073/pnas.1820226116 31101713 PMC6561300

[7] Mayer ML, Westbrook GL, Guthrie PB. Voltage-dependent block by Mg2+ of NMDA responses in spinal cord neurones. Nature. 1984. doi: 10.1038/309261a0 6325946

[8] Nowak L, Bregestovski P, Ascher P. et al. Magnesium gates glutamate-activated channels in mouse central neurones. Nature 307, 462–465 (1984). doi: 10.1038/307462a0 6320006

[9] Jahr CE, Stevens CF. Voltage Dependence of NMDA-Activated Predicted by Single-Channel Kinetics. J Neurosci. 1990;10: 3178–3182. doi: 10.1523/JNEUROSCI.10-09-03178.1990 1697902 PMC6570236

[10] Polsky A, Mel BW, Schiller J. Computational subunits in thin dendrites of pyramidal cells. Nat Neurosci. 2004;7: 621–7. doi: 10.1038/nn1253 15156147

[11] Niculescu D, Michaelsen-Preusse K, Güner Ü, van Dorland R, Wierenga CJ, Lohmann C. A BDNF-Mediated Push-Pull Plasticity Mechanism for Synaptic Clustering. Cell Rep. 2018;24: 2063–2074. doi: 10.1016/j.celrep.2018.07.073 30134168

[12] Kleindienst T, Winnubst J, Roth-Alpermann C, Bonhoeffer T, Lohmann C. Activity-dependent clustering of functional synaptic inputs on developing hippocampal dendrites. Neuron. 2011;72: 1012–1024. doi: 10.1016/j.neuron.2011.10.015 22196336

[13] Breton-Provencher V, Coté D, Saghatelyan A. Activity of the principal cells of the olfactory bulb promotes a structural dynamic on the distal dendrites of immature adult-born granule cells via activation of NMDA receptors. J Neurosci. 2014;34: 1748–1759. doi: 10.1523/JNEUROSCI.3013-13.2014 24478357 PMC6827585

[14] Takahashi N, Kitamura K, Matsuo N, Mayford M, Kano M, Matsuki N, et al. Locally Synchronized Synaptic Inputs. Science (80-). 2012;335: 353–356. doi: 10.1126/science.1210362 22267814

[15] Caroni P, Donato F, Muller D. Structural plasticity upon learning: regulation and functions. Nat Rev Neurosci. 2012;13: 478–490. doi: 10.1038/nrn3258 22714019

[16] Trachtenberg JT, Chen BE, Knott GW, Feng G, Sanes JR, Egbert Welker, et al. Long-term in vivo imaging of experience-dependent synaptic plasticity in adult cortex. Nature. 2002;420: 789–794. doi: 10.1038/nature01273 12490942

[17] Yang G, Pan F, Gan WB. Stably maintained dendritic spines are associated with lifelong memories. Nature. 2009;462: 920–924. doi: 10.1038/nature08577 19946265 PMC4724802

[18] Elston GN, Fujita I. Pyramidal cell development: Postnatal spinogenesis, dendritic growth, axon growth, and electrophysiology. Front Neuroanat. 2014;8: 1–20. doi: 10.3389/fnana.2014.00001 25161611 PMC4130200

[19] McBride TJ, Rodriguez-Contreras A, Trinh A, Bailey R, DeBello WM. Learning drives differential clustering of axodendritic contacts in the barn owl auditory system. J Neurosci. 2008;28: 6960–6973. doi: 10.1523/JNEUROSCI.1352-08.2008 18596170 PMC2581896

[20] Lu J, Zuo Y. Clustered structural and functional plasticity of dendritic spines. Brain Res Bull. 2017;129: 18–22. doi: 10.1016/j.brainresbull.2016.09.008 27637453 PMC5554072

[21] El-Boustani S, Ip JPK, Breton-Provencher V, Knott GW, Okuno H, Bito H, et al. Locally coordinated synaptic plasticity of visual cortex neurons in vivo. Science (80-). 2018;360: 1349–1354. doi: 10.1126/science.aao0862 29930137 PMC6366621

[22] Winnubst J, Cheyne JE, Niculescu D, Lohmann C. Spontaneous Activity Drives Local Synaptic Plasticity InVivo. Neuron. 2015;87: 399–411. doi: 10.1016/j.neuron.2015.06.029 26182421

[23] Kasthuri N, Jeffrey K, Berger DR, Priebe CE, Pfister H, Kasthuri N, et al. Saturated Reconstruction of a Volume of Neocortex Resource Saturated Reconstruction of a Volume of Neocortex. 2015; 648–661. doi: 10.1016/j.cell.2015.06.054 26232230

[24] Bartol TM, Bromer C, Kinney J, Chirillo MA, Bourne JN, Harris KM, et al. Nanoconnectomic upper bound on the variability of synaptic plasticity. Elife. 2015;4: 1–18. doi: 10.7554/eLife.10778 26618907 PMC4737657

[25] Motta A, Berning M, Boergens KM, Staffler B, Beining M, Loomba S, et al. Dense connectomic reconstruction in layer 4 of the somatosensory cortex. 2019;3134. doi: 10.1126/science.aay3134 31649140

[26] Fares T, Stepanyants A. Cooperative synapse formation in the neocortex. 2009;2009. 10.1073/pnas.0813265106 PMC2738618 19805321

[27] Jones TA, Klintsova AY, Kilman VL, Sirevaag AM, Greenough WT. Induction of multiple synapses by experience in the visual cortex of adult rats. Neurobiol Learn Mem. 1997;68: 13–20. doi: 10.1006/nlme.1997.3774 9195585

[28] Mel BW. The clusteron: Toward a simple abstraction for a complex neuron. Nips. 1991; 35–42.

[29] Kastellakis G, Cai DJ, Mednick SC, Silva AJ, Poirazi P. Synaptic clustering within dendrites: An emerging theory of memory formation. Progress in Neurobiology. Elsevier Ltd; 2015. pp. 19–35. doi: 10.1016/j.pneurobio.2014.12.002 PMC4361279 25576663

[30] Druckmann S, Feng L, Lee B, Yook C, Zhao T, Magee JC, et al. Structured Synaptic Connectivity between Hippocampal Regions. Neuron. 2014;81: 629–640. doi: 10.1016/j.neuron.2013.11.026 24412418

[31] DeBello WM, McBride TJ, Nichols GS, Pannoni KE, Sanculi D, Totten DJ. Input clustering and the microscale structure of local circuits. Front Neural Circuits. 2014;8: 112. doi: 10.3389/fncir.2014.00112 25309336 PMC4162353

[32] Wilson DE, Whitney DE, Scholl B, Fitzpatrick D. Orientation selectivity and the functional clustering of synaptic inputs in primary visual cortex. Nat Neurosci. 2016;19: 1003–1009. doi: 10.1038/nn.4323 27294510 PMC5240628

[33] Kerlin A, Boaz M, Flickinger D, Maclennan BJ, Dean MB, Davis C, et al. Functional clustering of dendritic activity during decision-making. Elife. 2019;8. doi: 10.7554/eLife.46966 31663507 PMC6821494

[34] Minsky ML, Papert S. Perceptrons. MIT Press. 1969. doi: 10.1364/AO.8.001341

[35] Koch C. Biophysics of Computation: Information Processing in Single Neurons (Computational Neuroscience Series). USA: Oxford University Press, Inc.; 2004.

[36] LeCun Y, Cortes C, Burges CJ. MNIST handwritten digit database. ATT Labs [Online] Available http://yann.lecun.com/exdb/mnist. 2010;2. doi: 10.1186/gb-2010-11-12-r123

[37] Duan K, Keerthi SS, Chu W, Shevade SK, Poo AN. Multi-category Classification by Soft-Max Combination of Binary Classifiers. In: Windeatt T, Roli F, editors. Multiple Classifier Systems. Berlin, Heidelberg: Springer Berlin Heidelberg; 2003. pp. 125–134.

[38] Kingma DP, Ba JL. Adam: A method for stochastic optimization. 3rd Int Conf Learn Represent ICLR 2015—Conf Track Proc. 2015; 1–15.

[39] Rumelhart DE, Hinton GE, Williams RJ. Learning representations by back-propagating errors. Nature. 1986;323: 533–536. doi: 10.1038/323533a0

[40] Poirazi P, Mel BW. Impact of active dendrites and structural plasticity on the memory capacity of neural tissue. Neuron. 2001;29: 779–796. doi: 10.1016/s0896-6273(01)00252-5 11301036

[41] Poirazi P, Brannon T, Mel BW. Pyramidal Neuron as Two-Layer Neural Network. Neuron. 2003;37: 989–999. doi: 10.1016/s0896-6273(03)00149-1 12670427

[42] Poirazi P, Mel BW. Towards the memory capacity of neurons with active dendrites. Neurocomputing. 1999;26–27: 237–245. doi: 10.1016/S0925-2312(99)00078-8

[43] Gidon A, Zolnik TA, Fidzinski P, Bolduan F, Papoutsi A, Poirazi P, et al. Dendritic action potentials and computation in human layer 2/3 cortical neurons. Science (80-). 2020;367: 83–87. doi: 10.1126/science.aax6239 31896716

[44] Jones IS, Kording KP. Can Single Neurons Solve MNIST? The Computational Power of Biological Dendritic Trees. arXiv. 2020.

[45] Schiess M, Urbanczik R, Senn W. Somato-dendritic Synaptic Plasticity and Error-backpropagation in Active Dendrites. PLoS Comput Biol. 2016;12: 1–18. doi: 10.1371/journal.pcbi.1004638 26841235 PMC4739747

[46] Urbanczik R, Senn W. Learning by the Dendritic Prediction of Somatic Spiking. Neuron. 2014;81: 521–528. doi: 10.1016/j.neuron.2013.11.030 24507189

[47] Hawkins J, Ahmad S. Why Neurons Have Thousands of Synapses, a Theory of Sequence Memory in Neocortex. Front Neural Circuits. 2016;10: 23. doi: 10.3389/fncir.2016.00023 27065813 PMC4811948

[48] Moldwin T, Segev I. Perceptron Learning and Classification in a Modeled Cortical Pyramidal Cell. Front Comput Neurosci. 2020;14: 1–13. doi: 10.3389/fncom.2020.00001 32390819 PMC7193948

[49] Mel BW. NMDA-Based Pattern Discrimination in a Modeled Cortical Neuron. Neural Comput. 1992;4: 502–517. doi: 10.1162/neco.1992.4.4.502

[50] Gilson M, Dahmen D, Insabato A, Helias M. The covariance perceptron: A new framework for classification and processing of time series in recurrent neural networks. bioRxiv. 2019; 1–37. doi: 10.1101/562546 PMC7595646 33044953

[51] Chen JL, Villa KL, Cha JW, So PTCC, Kubota Y, Nedivi E. Clustered Dynamics of Inhibitory Synapses and Dendritic Spines in the Adult Neocortex. Neuron. 2012;74: 361–373. doi: 10.1016/j.neuron.2012.02.030 22542188 PMC3340582

[52] Rall W. Distinguishing theoretical synaptic potentials computed for different soma-dendritic distributions of synaptic input. J Neurophysiol. 1967;30: 1138–1168. doi: 10.1152/jn.1967.30.5.1138 6055351

[53] Rall W. Branching dendritic trees and motoneuron membrane resistivity. Exp Neurol. 1959. doi: 10.1016/0014-4886(59)90046-9 14435979

[54] Rumsey CC, Abbott LF. Synaptic Democracy in Active Dendrites. J Neurophysiol. 2006;96: 2307–2318. doi: 10.1152/jn.00149.2006 16837665

[55] Magee JC, Cook EP. Somatic EPSP amplitude is independent of synapse location in hippocampal pyramidal neurons. Nat Neurosci. 2000;3: 895–903. doi: 10.1038/78800 10966620

[56] Komendantov AO, Ascoli GA. Dendritic excitability and neuronal morphology as determinants of synaptic efficacy. J Neurophysiol. 2009;101: 1847–1866. doi: 10.1152/jn.01235.2007 19176614 PMC2695639

[57] Branco T, Häusser M. The single dendritic branch as a fundamental functional unit in the nervous system. Curr Opin Neurobiol. 2010;20: 494–502. doi: 10.1016/j.conb.2010.07.009 20800473

[58] Losonczy A, Magee JC. Integrative Properties of Radial Oblique Dendrites in Hippocampal CA1 Pyramidal Neurons. Neuron. 2006;50: 291–307. doi: 10.1016/j.neuron.2006.03.016 16630839

[59] Harvey CD, Yasuda R, Zhong H, Svoboda K. The Spread of Ras Activity. Science (80-). 2008;321: 136–140. doi: 10.1126/science.1159675 18556515 PMC2745709

[60] Harvey CD, Svoboda K. Locally dynamic synaptic learning rules in pyramidal neuron dendrites. 2007;450. doi: 10.1038/nature06416 18097401 PMC3425382

[61] Mendez P, Garcia-Segura LM, Muller D. Estradiol promotes spine growth and synapse formation without affecting pre-established networks. Hippocampus. 2011;21: 1263–1267. doi: 10.1002/hipo.20875 20928832

[62] Murphy DD, Cole NB, Segal M. Brain-derived neurotrophic factor mediates estradiol-induced dendritic spine formation in hippocampal neurons. Proc Natl Acad Sci U S A. 1998;95: 11412–11417. doi: 10.1073/pnas.95.19.11412 9736750 PMC21656

[63] Stein IS, Zito K. Dendritic Spine Elimination: Molecular Mechanisms and Implications. Neuroscientist. 2019;25: 27–47. doi: 10.1177/1073858418769644 29716431 PMC6167191

[64] Bliss TVP, Collingridge GL. A synaptic model of memory: Long-term potentiation in the hippocampus. Nature. Nature Publishing Group; 1993. pp. 31–39. doi: 10.1038/361031a0 8421494

[65] Patterson M, Yasuda R. Signalling pathways underlying structural plasticity of dendritic spines. British Journal of Pharmacology. Wiley-Blackwell; 2011. pp. 1626–1638. doi: 10.1111/j.1476-5381.2011.01328.x PMC3166652 21410464

[66] Nägerl UV, Eberhorn N, Cambridge SB, Bonhoeffer T. Bidirectional activity-dependent morphological plasticity in hippocampal neurons. Neuron. 2004;44: 759–767. doi: 10.1016/j.neuron.2004.11.016 15572108

[67] Harvey CD, Svoboda K. Locally dynamic synaptic learning rules in pyramidal neuron dendrites. Nature. 2007;450: 1195–1200. doi: 10.1038/nature06416 18097401 PMC3425382

[68] Schultz W. Predictive Reward Signal of Dopamine Neurons. J Neurophysiol. 1998;80: 1–27. doi: 10.1152/jn.1998.80.1.1 9658025

[69] Lisman J, Grace AA, Duzel E. A neoHebbian framework for episodic memory; role of dopamine-dependent late LTP. Trends Neurosci. 2011;34: 536–547. doi: 10.1016/j.tins.2011.07.006 21851992 PMC3183413

[70] Cohen JD, Braver TS, Brown JW. Computational perspectives on dopamine function in prefrontal cortex. Current Opinion in Neurobiology. Elsevier Ltd; 2002. pp. 223–229. doi: 10.1016/s0959-4388(02)00314-8 12015241

[71] Jadi MP, Behabadi BF, Poleg-Polsky A, Schiller J, Mel BW. An augmented two-layer model captures nonlinear analog spatial integration effects in pyramidal neuron dendrites. Proc IEEE. 2014;102: 782–798. doi: 10.1109/JPROC.2014.2312671 25554708 PMC4279447

[72] Behabadi BF, Polsky A, Jadi M, Schiller J, Mel BW. Location-dependent excitatory synaptic interactions in pyramidal neuron dendrites. PLoS Comput Biol. 2012. doi: 10.1371/journal.pcbi.1002599 22829759 PMC3400572

[73] Jadi M, Polsky A, Schiller J, Mel BW. Location-dependent effects of inhibition on local spiking in pyramidal neuron dendrites. PLoS Comput Biol. 2012. doi: 10.1371/journal.pcbi.1002550 22719240 PMC3375251

[74] Hiratani N, Fukai T. Redundancy in synaptic connections enables neurons to learn optimally. Proc Natl Acad Sci U S A. 2018;115: E6871–E6879. doi: 10.1073/pnas.1803274115 29967182 PMC6055135

[75] Roelants P. Logistic classification with cross-entropy. [cited 14 Mar 2021]. Available: https://peterroelants.github.io/posts/cross-entropy-logistic/

[76] Abadi M, Barham P, Chen J, Chen Z, Davis A, Dean J, et al. TensorFlow: A system for large-scale machine learning. Proceedings of the 12th USENIX Symposium on Operating Systems Design and Implementation, OSDI 2016. 2016.

[77] Pedregosa F, Varoquaux G, Gramfort A, Michel V, Thirion B, Grisel O, et al. Scikit-learn: Machine learning in Python. J Mach Learn Res. 2011.

[78] Liu DC, Nocedal J. On the limited memory BFGS method for large scale optimization. Math Program. 1989;45: 503–528. doi: 10.1007/BF01589116

[79] Polyak BT, Juditsky AB. Acceleration of stochastic approximation by averaging. SIAM J Control Optim. 1992;30: 838–855. doi: 10.1137/0330046

