# Supplementary material for: The gradient clusteron: A model neuron that learns to solve classification tasks via dendritic nonlinearities, structural plasticity, and gradient descent
Source: PLoS Comput Biol. 2021 May 24;17(5):e1009015. doi: 10.1371/journal.pcbi.1009015 (PMC8177649; doi:10.1371/journal.pcbi.1009015)
Supplement: S1 Text — (PDF) [file pcbi.1009015.s006.pdf]

S1. Explanation of signs and directionality in the location update rule.

Given the location update rule for synapse i (Eq. 7 in **Results**):

$$\Delta l_i = -\eta_L(\hat{y} - y)\sum_{j=1}^{N}(l_j - l_i)F_{ij}w_i x_i w_j x_j$$

We would like to demonstrate how the update would work on a simple example. Consider a dendrite with two synapses. We first consider the effect of the update rule on the left synapse, so we designate the left synapse as synapse i. Because $(l_i - l_i) = 0$, the only contributing term in the summation is that of the right synapse, which we designate as synapse j.

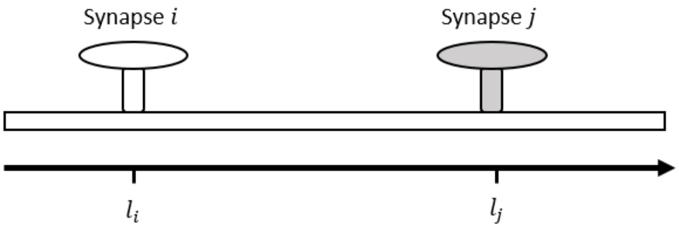

The update rule is thus:

$$\Delta l_i = -\eta_L(\hat{y} - y)(l_j - l_i)F_{ij}w_i x_i w_j x_j$$

Because synapse i is to the left of synapse j, synapse i is "attracted" to synapse j if the update rule pushes synapse i to the right (i.e. $\Delta l_i > 0$) and "repelled" from synapse j if the update rule pushes synapse i to the left (i.e. $\Delta l_i < 0$). To determine the sign of Δl_i for different conditions (positive/negative class, same/opposite-sign weighted inputs) we multiply the signs of each factor in the update rule. Note that because synapse i is to the left of synapse j, $(l_j - l_i)$ is positive.

**Target class (y)**	$sign(w_i x_i) = sign(w_j x_j)$**?**	**-η_L**	$(\hat{y} - y)$	$(l_j - l_i)$	F_{ij}	$w_i x_i * w_j x_j$	Δl_i
Positive class ($y = 1$)	Yes	-	-	+	+	+	+
Negative class ($y = 0$)	Yes	-	+	+	+	+	-
Positive class ($y = 1$)	No	-	-	+	+	-	-
Negative class ($y = 0$)	No	-	+	+	+	-	+

Now we consider the effect on the right synapse. We therefore designate the synapse on the right as synapse i. (Note that these are the same synapses as before, we just use the designation i for the synapse that is being updated and the designation j for the other synapse.)

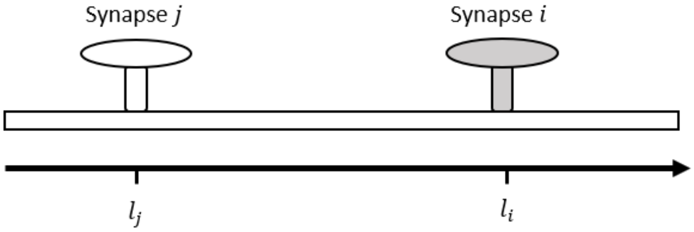

Now “attraction” occurs if synapse i moves to the left (i.e. $\Delta l_i < 0$), and “repulsion” occurs if synapse i moves to the right (i.e. $\Delta l_i > 0$). We have the same table for Δl_i as before, except now $(l_j - l_i)$ is negative, flipping the sign of Δl_i.

Target class (y)	$\boldsymbol{sign(w_i x_i) = sign(w_j x_j)}$**?**	$-\eta_L$	$(\hat{y} - y)$	$(l_j - l_i)$	F_{ij}	$w_i x_i * w_j x_j$	Δl_i
Positive class ($y = 1$)	Yes	-	-	-	+	+	-
Negative class ($y = 0$)	Yes	-	+	-	+	+	+
Positive class ($y = 1$)	No	-	-	-	+	-	+
Negative class ($y = 0$)	No	-	+	-	+	-	-

The net result is that both synapses are either attracted or repelled to each other, depending on the target class and the signs of the weighted inputs, as described in the **Results** section of the paper.
