# Supplementary material for: The gradient clusteron: A model neuron that learns to solve classification tasks via dendritic nonlinearities, structural plasticity, and gradient descent
Source: PLoS Comput Biol. 2021 May 24;17(5):e1009015. doi: 10.1371/journal.pcbi.1009015 (PMC8177649; doi:10.1371/journal.pcbi.1009015)
Supplement: S2 Text — (PDF) [file pcbi.1009015.s007.pdf]

S2 Text. Results for multiple trial runs of the multivariate Gaussian task.

For the multivariate Gaussian task (Figure 3), to verify that there was an increase in activation (for the positive class task, Figure 3C) or decrease in average activation (for the negative class task, Figure 3D) after learning, we ran 100 trials of the learning algorithm for each task. In each trial, the G-clusterons were tested on a test set of 1000 patterns randomly sampled from the multivariate Gaussian distribution (Figure 3A) before and after learning.

The plots below show the paired mean activations (circles) and standard deviations (error bars) of the activations before and after learning for each trial on the test set. The difference in activation before and after learning was quantified by averaging over the signed d' statistic for each trial, where the signed d' in this case is defined as $d' = \frac{\mu_f - \mu_i}{\sqrt{\frac{1}{2}(\sigma_f^2 + \sigma_i^2)}}$ where μ_i and σ_i are the mean and standard deviation of the average synaptic activations initially (before learning), and μ_f and σ_f are the mean and standard deviation of the average synaptic activations after the final epoch (after learning).

The average activation on the positive class task before learning was 0.86, and after learning was 2.95. For the negative class task, average activation before learning was 2.03, and after learning was 0.44.

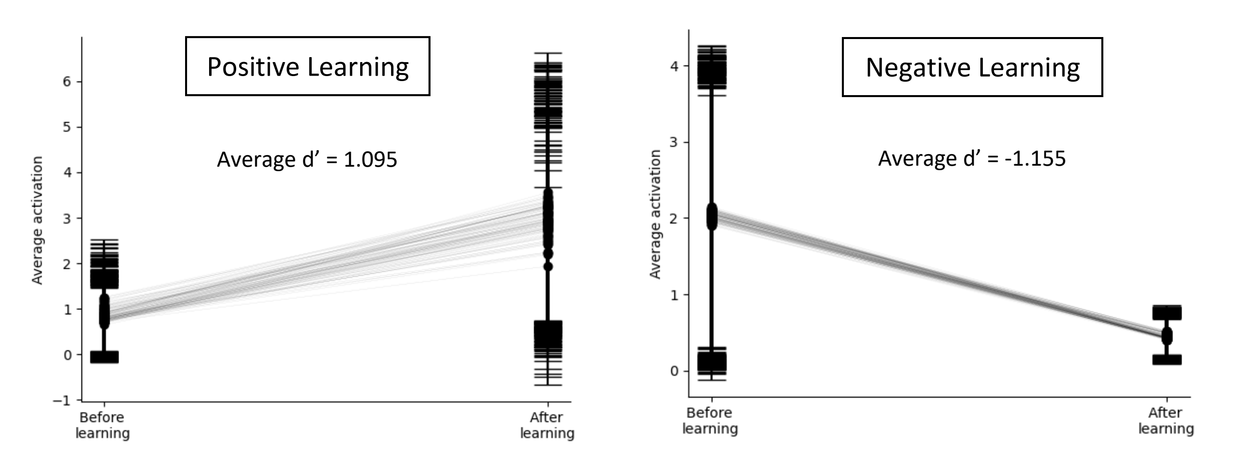

Figure S2 Text. Paired sample plot for multivariate Gaussian example
Test set outputs for 100 trials of the multivariate Gaussian task, for both the positive class task (left) and the negative class task (right). Shown are the paired mean activations (circles) and standard deviations (error bars) before and after learning for each trial on the test set.
