# Supplementary material for: The gradient clusteron: A model neuron that learns to solve classification tasks via dendritic nonlinearities, structural plasticity, and gradient descent
Source: PLoS Comput Biol. 2021 May 24;17(5):e1009015. doi: 10.1371/journal.pcbi.1009015 (PMC8177649; doi:10.1371/journal.pcbi.1009015)
Supplement: S3 Text — (PDF) [file pcbi.1009015.s008.pdf]

S3. Proof that the G-clusteron can approximate a linear classifier

To prove that every linear classifier of the form $h(x) = \sum_{i=1}^{N} w_i x_i - b$ can be approximated to arbitrary precision by a weighted G-clusteron (with any arrangement of synaptic locations) of the form $h^*(x) = \sum_{i=0}^{N} w_i^* x_i \sum_{j=0}^{N} F_{ij} w_j^* x_j - b^*$ with the inclusion of a single "bias synapse" x_0 ($x_0 = 1$ for all input patterns, this is distinct from the zeroth-order bias term b^*), we observe that:

$$h^*(x) = w_0^{*2} x_0^2 + \sum_{i=1}^{N} 2F_{i0} w_i^* x_i w_0^* x_0 + \sum_{i=1}^{N} w_i^* x_i \sum_{j=1}^{N} F_{ij} w_j^* x_j - b^* \qquad \text{(S3.1)}$$

Because $x_0 = 1$, this expression simplifies to:

$$h^*(x) = w_0^{*2} + \sum_{i=1}^{N} 2F_{i0} w_i^* x_i w_0^* + \sum_{i=1}^{N} w_i^* x_i \sum_{j=1}^{N} F_{ij} w_j^* x_j - b^* \qquad \text{(S3.2)}$$

We set the weights of the G-clusteron (other than the bias synapse weight w_0^*):

$$w_i^* = \frac{w_i}{2F_{i0} w_0^*} \; for\ i > 1 \qquad \text{(S3.3)}$$

And we set the bias of the G-clusteron as:

$$b^* = w_0^{*2} + b \qquad \text{(S3.4)}$$

We therefore have:

$$\begin{aligned} h^*(x) &= \sum_{i=1}^{N} w_i x_i + \sum_{i=1}^{N} \frac{w_i}{2F_{i0} w_0^*} x_i \sum_{j=1}^{N} \frac{F_{ij} w_j}{2F_{j0} w_0^*} x_j - b \\ &= \sum_{i=1}^{N} w_i x_i - b + \frac{1}{w_0^{*2}} \sum_{i=1,j=1}^{N} \frac{F_{ij} w_i w_j}{4F_{i0} F_{j0}} x_i x_j \end{aligned} \qquad \text{(S3.5)}$$

If we set w_0^* to be a very large number such that:

$$w_0^* \gg 1 \qquad \text{(S3.6)}$$

The final term for Eq. S3.5 approaches 0, and we therefore have:

$$h(x) \approx \sum_{i=1}^{N} w_i x_i - b \qquad \text{(S3.7)}$$

Note that this approximation can be obtained for any arrangement of the synaptic locations in the G-clusteron as long as the weights are appropriately set as in Eq. S3.3.
