# Supplementary material for: The gradient clusteron: A model neuron that learns to solve classification tasks via dendritic nonlinearities, structural plasticity, and gradient descent
Source: PLoS Comput Biol. 2021 May 24;17(5):e1009015. doi: 10.1371/journal.pcbi.1009015 (PMC8177649; doi:10.1371/journal.pcbi.1009015)
Supplement: S4 Text — (PDF) [file pcbi.1009015.s009.pdf]

S4. Extended derivation of location update rule

To derive the location update rule, we need to find the derivative of the raw output of the G-clusteron h with respect to an arbitrary location l_k. (In **Results** and **Methods** we used the notation $\frac{\delta h}{\delta l_i}$; here we use the notation $\frac{\delta h}{\delta l_k}$ to avoid confusion with the summation indices):

$$\frac{\delta h}{\delta l_k} = \frac{\delta}{\delta l_k}\left(\sum_{i=1}^{N} a_i - b\right) \tag{S4.1}$$

$$= \frac{\delta}{\delta l_k}\left(\sum_{i=1}^{N} w_i x_i \sum_{j=1}^{N} e^{\frac{-\left(l_i - l_j\right)^2}{r}} w_j x_j - b\right) \tag{S4.2}$$

We break this expression into four terms reflecting the four possible conditions: $(i = k, j = k), (i \neq k, j \neq k), (i = k, j \neq k), (i \neq k, j = k)$

$$= \frac{\delta}{\delta l_k}\left(w_k x_k w_k x_k + \sum_{i=1,i\neq k}^{N} w_i x_i \sum_{j=1,j\neq k}^{N} e^{\frac{-\left(l_i - l_j\right)^2}{r}} w_j x_j + w_k x_k \sum_{j=1,j\neq k}^{N} e^{\frac{-\left(l_k - l_j\right)^2}{r}} w_j x_j + \sum_{i=1,i\neq k}^{N} w_i x_i\, e^{\frac{-(l_i - l_k)^2}{r}} w_k x_k - b\right) \tag{S4.3}$$

We calculate the derivative of each term separately:

Term	$\frac{\delta}{\delta l_k}$
$w_k x_k w_k x_k$	0
$\sum_{i=1,i\neq k}^{N} w_i x_i \sum_{j=1,j\neq k}^{N} e^{\frac{-\left(l_i - l_j\right)^2}{r}} w_j x_j$	0
$w_k x_k \sum_{j=1,j\neq k}^{N} e^{\frac{-\left(l_k - l_j\right)^2}{r}} w_j x_j$	$-\frac{2}{r} w_k x_k \sum_{j=1,j\neq k}^{N} (l_k - l_j) e^{\frac{-\left(l_k - l_j\right)^2}{r}} w_j x_j$
$\sum_{i=1,i\neq k}^{N} w_i x_i\, e^{\frac{-(l_i - l_k)^2}{r}} w_k x_k$	$\frac{2}{r} \sum_{i=1,i\neq k}^{N} w_i x_i\, (l_i - l_k) e^{\frac{-(l_i - l_k)^2}{r}} w_k x_k$

Noting that the expressions in the last two rows are equivalent, summing the derivatives of all four terms gives us:

$$\frac{4}{r} w_k x_k \sum_{j=1,j\neq k}^{N} w_j x_j \left(l_j - l_k\right) e^{\frac{-\left(l_j - l_k\right)^2}{r}} \tag{S4.4}$$

Noting that when $j = k$, $w_j x_j \left(l_j - l_k\right) e^{\frac{-\left(l_j - l_k\right)^2}{r}} = 0$, we have:

$$\frac{\delta h}{\delta l_k} = \frac{4}{r} \sum_{j=1}^{N} \left(l_j - l_k\right) e^{\frac{-\left(l_j - l_k\right)^2}{r}} w_k x_k w_j x_j \tag{S4.5}$$
