# Supplementary material for: The gradient clusteron: A model neuron that learns to solve classification tasks via dendritic nonlinearities, structural plasticity, and gradient descent
Source: PLoS Comput Biol. 2021 May 24;17(5):e1009015. doi: 10.1371/journal.pcbi.1009015 (PMC8177649; doi:10.1371/journal.pcbi.1009015)
Supplement: S5 Text — (PDF) [file pcbi.1009015.s010.pdf]

**S5 - Extended derivation of weight update rule**

As with the location rule (Eq. S4.1), we need to find the derivative of the raw output of the G-clusteron $h$ with respect to an arbitrary weight $w_k$:

$$\frac{\delta h}{\delta w_k} = \frac{\delta}{\delta w_k}\left(\sum_{i=1}^{N} a_i - b\right) \tag{S5.1}$$

As with the location rule, we break $h$ up into four terms and calculate the derivatives separately:

| Term | $\frac{\delta}{\delta w_k}$ |
|---|---|
| $w_k x_k w_k x_k$ | $2x_k^2 w_k$ |
| $\sum_{i=1,i\neq k}^{N} w_i x_i \sum_{j=1,j\neq k}^{N} e^{\frac{-\left(l_i - l_j\right)^2}{r}} w_j x_j$ | 0 |
| $w_k x_k \sum_{j=1,j\neq k}^{N} e^{\frac{-\left(l_k - l_j\right)^2}{r}} w_j x_j$ | $x_k \sum_{j=1,j\neq k}^{N} e^{\frac{-\left(l_k - l_j\right)^2}{r}} w_j x_j$ |
| $\sum_{i=1,i\neq k}^{N} w_i x_i\, e^{\frac{-(l_i - l_k)^2}{r}} w_k x_k$ | $\sum_{i=1,i\neq k}^{N} w_i x_i\, e^{\frac{-(l_i - l_k)^2}{r}} x_k$ |

Noting that the expressions in the last two rows are equivalent, summing the derivatives of all four terms gives us:

$$= 2x_k^2 w_k + \sum_{j=1,j\neq k}^{N} 2e^{\frac{-\left(l_k - l_j\right)^2}{r}} w_j x_j x_k \tag{S5.2}$$

Noting that when $j = k$:

$$2e^{\frac{-\left(l_k - l_j\right)^2}{r}} w_j x_j x_k = 2x_k^2 w_k \tag{S5.3}$$

We have:

$$\frac{\delta h}{\delta w_k} = 2x_k \sum_{j=1}^{N} e^{\frac{-\left(l_k - l_j\right)^2}{r}} w_j x_j = 2\frac{a_k}{w_k} \tag{S5.4}$$
